# In situ formation of catalytically active graphene in ethylene photo-epoxidation

**DOI:** 10.1038/s41467-018-05352-9

**Published:** 2018-08-03

**Authors:** Xueqiang Zhang, Gayatri Kumari, Jaeyoung Heo, Prashant K. Jain

**Affiliations:** 1 Department of Chemistry, University of Illinois at Urbana-Champaign, 600 S Mathews Avenue, Urbana, IL 61801 USA; 2 Department of Materials Science and Engineering, University of Illinois at Urbana-Champaign, 1304 W Green Street, Urbana, IL 61801 USA; 3 Materials Research Laboratory, University of Illinois at Urbana-Champaign, 104 S Goodwin Avenue, Urbana, IL 61801 USA

## Abstract

Ethylene epoxidation is used to produce 2 × 10^7^ ton per year of ethylene oxide, a major feedstock for commodity chemicals and plastics. While high pressures and temperatures are required for the reaction, plasmonic photoexcitation of the Ag catalyst enables epoxidation at near-ambient conditions. Here, we use surface-enhanced Raman scattering to monitor the plasmon excitation-assisted reaction on individual sites of a Ag nanoparticle catalyst. We uncover an unconventional mechanism, wherein the primary step is the photosynthesis of graphene on the Ag surface. Epoxidation of ethylene is then promoted by this photogenerated graphene. Density functional theory simulations point to edge defects on the graphene as the sites for epoxidation. Guided by this insight, we synthesize a composite graphene/Ag/α-Al_2_O_3_ catalyst, which accomplishes ethylene photo-epoxidation under ambient conditions at which the conventional Ag/α-Al_2_O_3_ catalyst shows negligible activity. Our finding of in situ photogeneration of catalytically active graphene may apply to other photocatalytic hydrocarbon transformations.

## Abstract (web summary)

In situ studies under working conditions are important in atomic-level elucidation, design, and optimization of industrially relevant catalysts. Here, the authors report an in situ study of an Ag ethylene photo-epoxidation catalyst using surface enhanced Raman scattering, which uncovers an unconventional mechanism.

## Introduction

Heterogeneous catalysts facilitate the economical production of commodity and fine chemicals, but their workings are often complex and shrouded in mystery^1,2^. Although the nature of the active site of the catalyst is often deducible from electronic structure simulations or ultrahigh vacuum single-crystal studies, but under realistic working conditions, the catalyst can be appreciably different in structure and mechanism from the idealization. Moreover, under reaction conditions, the catalytically active phase can involve a dynamically changing surface structure and/or composition, which is not reflected in a steady-state picture. In situ studies under working conditions are therefore of central importance in atomic-level elucidation, design, and optimization of industrially relevant catalysts.

The epoxidation of ethylene (C_2_H_4_) to ethylene oxide (EO), a major feedstock for several organic chemicals and plastics, is practiced on a massive scale of ~2 × 10^7^ tons^3^ and tens of billions of dollars worth of annual production^4^. The epoxidation reaction is catalyzed by an α-Al_2_O_3_-supported Ag-based catalyst under high pressure (10–30 bar) and high temperature (200–300 °C) conditions^5^, a process that accomplishes high selectivity for EO in the face of the competing full oxidation of C_2_H_4_ to CO_2_. However, accomplishment of C_2_H_4_ epoxidation at moderate pressures and temperatures is desirable^6,7^ from the point of view of reducing heat energy input, enhancing energy efficiency, mitigating thermal damage and deactivation of the catalyst, and further enhancing EO selectivity by reducing high-temperature-favored processes such as isomerization of EO to acetaldehyde^8^ and full oxidation to CO_2_. Recently, visible-light photoexcitation of plasmon-resonant Ag nanoparticles (NPs) has been shown to be effective at achieving C_2_H_4_ epoxidation at less elevated temperatures and pressures^6,7,9^.

With the aim of understanding how C_2_H_4_ epoxidation is catalyzed under visible-light excitation and ambient atmosphere conditions, we monitor the process on a supported Ag NP photocatalyst. Using in situ surface-enhanced Raman scattering (SERS), we interrogate surface chemical events occurring in real time on individual domains of the photocatalyst. The interrogation reveals an active phase and working mechanism of the photocatalyst deviating markedly from the presupposed picture of a pristine metal surface. The primary step in the photocatalysis is the dynamic photosynthesis of graphenic carbon from the C_2_H_4_ precursor. The in situ photogenerated graphene serves to actively promote further C_2_H_4_ epoxidation. Armed with the insight that the active photocatalytic phase involves graphenic carbon and not just the pristine metal surface, we prepare a composite graphene/Ag/α-Al_2_O_3_ photocatalyst. This composite photocatalyst accomplishes epoxidation at ambient temperature and pressure conditions, where a conventional Ag/α-Al_2_O_3_ photocatalyst shows low activity. Our study provides an example of how realistic knowledge of the active form of the photocatalyst can lead to advances in an industrially important chemical process.

## Results and discussion

### SERS monitoring of photocatalytic C_2_H_4_ epoxidation on Ag NPs

**Fig. 1:**
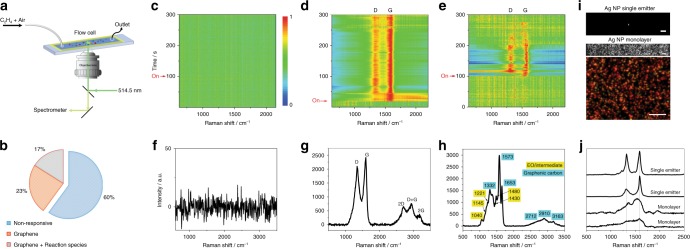
In situ SERS study of Ag NP-photocatalyzed C_2_H_4_ epoxidation. **a** Schematic of experimental setup, where a home-made reaction flow cell is mounted in an optical microscope for in situ SERS study. The SERS monitoring was carried out under the flow of C_2_H_4_ in air, which serves as the source of oxygen. **b** Pie chart of activity across 220 Ag NP emitters, showing three classes: non-responsive NP emitters (~60%), those exhibiting graphenic carbon formation (23%), and those showing graphenic carbon formation along with C_2_H_4_ oxidation species (17%). A time series of SERS spectra, under photocatalytic reaction conditions, is shown by a waterfall plot for representative cases of **c** a non-responsive Ag NP emitter, **d** a Ag NP emitter that shows the formation of only graphenic carbon, and **e** a Ag NP emitter showing formation of both graphenic carbon and reactive species related to C_2_H_4_ oxidation species. Panels **c**–**e** share the same color scale and *y*-axis title. SERS spectra were acquired continuously at a rate of 0.2 s per frame and are shown normalized. The instant when C_2_H_4_ flow was introduced (and sample refocused) is labeled as on. A representative unnormalized SERS spectrum (*t* = 160 s slice, *t* = 64 s slice, *t* = 240.2 s slice from water-fall plots in **c**, **d**, **e**, respectively) demonstrates **f** a non-responsive Ag NP emitter, **g** presence of graphenic carbon, indicated by SERS peaks associated with D, G, 2D, 2G, and D + G vibrational modes, and **h** presence of both graphenic carbon and reaction species, respectively. Each key vibrational mode is labeled with the peak wavenumber indicated in cm^−1^. Panels **f**–**h** and **j** share the same *y*-axis title. **i** Representative ×60 CCD images of a single laser-excited Ag NP emitter within the spectrograph slit (top, scale bar = 20 μm) and white light-illuminated wide-field region of a Ag NP monolayer (middle, scale bar = 20 μm), and a ×100 color camera dark-field scattering image of the Ag NP monolayer (bottom, scale bar = 10 μm). **j** Comparison of SERS spectra (single time-slice) obtained from a single Ag NP emitter (two representative cases) with those from a Ag NP monolayer (two representative cases), demonstrating the necessity for single-emitter-level spectroscopy. Spectra are shown stacked

The SERS monitoring was performed in a home-made reaction flow cell (Fig. 1a). The catalyst consisted of Ag NPs (Supplementary Figs. 1, 2) immobilized at a low area density on the SiO_2_ surface of the cell (Supplementary Fig. 3). A visible-wavelength laser served as the source for both photoactivation and SERS. SERS from individual emitters was monitored in real time with sub-second resolution under reaction conditions (C_2_H_4_ + ambient air + 514.5 nm laser excitation). Here, an emitter refers to a discrete light-absorbing, light-scattering domain, consisting of one or a few Ag NPs (Supplementary Fig. 3). The single-domain spatial resolution and high sensitivity of SERS allowed us to capture reactants, intermediates, and products stochastically formed on the catalyst surface (Fig. 1), which are otherwise undetectable in spatially averaged measurements, due to their short lifetimes on the surface and/or low steady-state concentrations.

Prior to the introduction of C_2_H_4_, no distinct SERS bands are seen from individual emitters (Fig. 1d, e), confirming the surface cleanliness of the catalyst. After C_2_H_4_ is introduced, distinct SERS bands are seen to emerge (Fig. 1d, e and Supplementary Fig. 4) in some cases, following an induction period. Nearly 40% of all individual emitters monitored (Fig. 1b) showed such dynamic chemical activity. The remaining ~60% of the emitters show non-responsive SERS spectra under C_2_H_4_ flow (Fig. 1c, f), possibly due to the absence of an electromagnetic field hotspot for ample SERS amplification (e.g., single Ag NPs in Supplementary Fig. 3) or due to a significantly oxidized, inactive Ag surface. We did not study this non-responsive class of emitters further, instead focusing our attention on the responsive sub-population.

The SERS bands observed under reaction conditions (i.e., C_2_H_4_ flow) are well-defined with peak maxima at ~1340 and ~1570 cm^−1^, which can be assigned, respectively, to the D and G bands of graphenic carbon. The presence of vibrational overtones at 2650 cm^−1^ (2D mode), 2890 cm^−1^ (D + G mode), and 3160 cm^−1^ (2G mode) further confirms the assignment of the SERS spectrum (Fig. 1g and Supplementary Fig. 5) to graphenic carbon^10–12^. High-resolution transmission electron microscopy (HRTEM) characterization, discussed later, provides visual evidence of the formed graphene. We confirm that C_2_H_4_ acts as the direct precursor for graphene generation from the results shown in Fig. 1d, e, where the emergence of graphene features is observed to be correlated with the introduction of C_2_H_4_. Thus, it appears that the condensation of C_2_H_4_ to graphene on the Ag surface is a primary step under the photo-epoxidation conditions.

As per recent reports, C_2_H_4_ monomers can undergo condensation reactions at high temperatures (e.g., 770–970 K) on transition metal surfaces (e.g., Rh (111) to form extended graphene^13)^. In the present case, the ambient temperature formation of graphenic carbon appears to be photoactivated by visible-light excitation of the plasmon-resonant Ag NPs. The graphene is formed from condensation of C_2_H_4_ monomers via a photo-driven process on the Ag NP surface. On the basis of recent plasmon-assisted bond-dissociation reactions^6,7,9^, we propose that the plasmon-excited Ag NP surface and/or surface-adsorbed O_2_ is involved in hot electron-assisted hydride abstraction from the C_2_H_4_. The resulting carbene species can polymerize to form graphene.

Such photosynthesis of graphene at ambient temperature ought to be contrasted from the formation of amorphous carbon or coking of catalyst surfaces known in high-temperature catalytic cracking processes^14,15^. In the latter processes, hydrocarbon reactants and/or products, particularly of the aromatic kind, strongly adsorb on the catalyst surface, where they gradually accumulate followed by chemical reactions to produce amorphous carbon deposits of low volatility, resulting in poisoning of the catalyst. Whereas surface carbon formation or coking is known to contaminate or poison the metal catalyst; the in situ generated graphenic carbon in our case appears to promote C_2_H_4_ photo-epoxidation, as described in the next section.

**Fig. 2:**
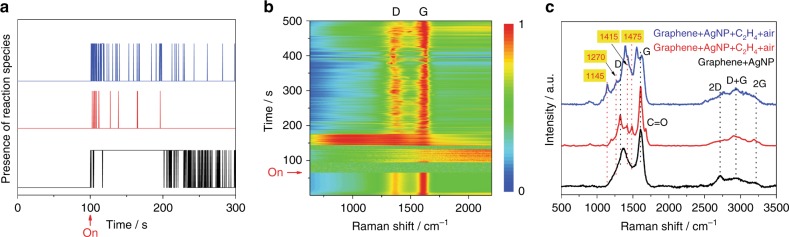
Graphenic carbon formed in situ catalyzes C_2_H_4_ epoxidation. **a** Digital reaction trajectory (presence of a reaction species vs. time) for a representative single Ag NP emitter under photocatalytic C_2_H_4_ oxidation conditions corresponding to the data in Fig. 1e. The SERS monitoring was carried out under the flow of C_2_H_4_ in air, which serves as the source of oxygen. The trajectories, shown stacked, demonstrate the correlation between the presence of graphenic carbon and the formation and detection of C_2_H_4_ oxidation reaction species, as also shown by additional examples in Supplementary Fig. 5. **b** Time series of SERS spectra, shown by a waterfall plot, for a single graphene-covered Ag NP emitter. Continuous SERS spectra were taken at a rate of 0.2 s per frame and are shown normalized. The instant when C_2_H_4_ flow was introduced is labeled as on. **c** Selected time slices from in situ SERS study of the single graphene-covered Ag NP emitter in (**b**). The SERS spectrum before start of C_2_H_4_ flow (black curve, *t* = 40 s slice) shows D, G, 2D, and D + G modes of graphene. The SERS spectra under C_2_H_4_ epoxidation conditions (red curve, *t* = 372 s and blue curve, *t* = 181.2 s) show formation of EO. Spectra are shown normalized and stacked with the key vibrational modes labeled with the peak wavenumbers indicated in cm^−1^

The 2D band of the in situ generated graphene (Fig. 1g and Supplementary Fig. 5) is significantly weaker in intensity compared to that for free-standing, pristine graphene (Supplementary Fig. 6). It is known that a suppressed 2D band is a signature of disruption in the electronic structure of graphene, like those caused by molecular adsorbates, lattice defects, and/or graphene–metal substrate electronic interactions^16–20^. Based on this known characteristic, the weak 2D band appears to be an indicator of the electronic contact of the in situ generated graphene fragment with the Ag NP surface, n-doping of the graphene by Ag, and/or unterminated bonds of the fragments. Also, it must be noted that the graphenic carbon bands (see Supplementary Table 1) can be assigned either to graphene or to graphene oxide, which can inter-change with each other under laser irradiation in ambient air: graphene can photo-oxidize into graphene oxide^21^ and the latter can be reduced back to graphene by laser excitation^22^, assisted by the light-absorbing Ag NP. The graphenic carbon bands show intermittency (Fig. 2a and Supplementary Figs. 4, 7), which may signify dynamic formation/breakdown of graphenic carbon fragments under laser excitation.

There have been past bulk SERS studies of thermally activated C_2_H_4_ epoxidation on Ag^23–25^, which have provided considerable insight into the surface intermediates and reactions involved in the catalysis. Under some conditions, the formation of surface carbon has been observed^26–28^; however, the identity of these carbonaceous species remained unresolved due to the broad and featureless nature of the observed vibrational modes. Our finding of crystalline carbon, specifically graphenic carbon, in photocatalytic epoxidation on Ag NPs is enabled by the high spatial (single micron-scale emitter) and temporal (200 ms) resolution of our SERS monitoring, whereby ensemble averaging is minimized. We demonstrate this by comparing SERS spectra from individual emitters of a well-dispersed Ag NP catalyst against those from monolayers of Ag NPs, under similar C_2_H_4_ photo-epoxidation conditions (Fig. 1i). The Ag NP monolayers yield broad carbon-related modes in SERS spectra, in contrast to the well-defined, signature graphene bands seen in the case of single-emitter SERS.

The formation of graphene on Ag NPs under visible-light excitation did not appear to be exclusive to one size of Ag NPs. Ag NPs of two markedly different sizes (average sizes of 28 and 53 nm as shown in Supplementary Fig. 1) showed the same behavior (Supplementary Fig. 5). Note that in our size selection, we were restricted to the 25–70 nm size range, because only Ag NPs in this range provide SERS enhancement factors large enough for single NP-level SERS imaging.

### Photogenerated graphene promotes C_2_H_4_ photo-epoxidation

Of the emitters that exhibit in situ formed graphene, nearly 40% (equivalent to 17% of the entire population, Fig. 1b) exhibit the formation of reaction species associated with C_2_H_4_ oxidation (Fig. 1e). As shown by the representative example in Fig. 1h and several others in Supplementary Fig. 5, selected SERS spectral frames show sharp peaks (labeled in Fig. 1h) co-existing with the graphenic carbon D and G bands. Based on literature (Supplementary Table 1) as well as our density functional theory (DFT) calculations (Table 1), these observed SERS peaks are found to be vibrational modes of reactive intermediates and products EO (1025, 1125, 1147, 1170, 1216, 1279, 1415, and 1475 cm^−1^) and CO_2_ (1283 and 1380 cm^−1^) of C_2_H_4_ epoxidation.

**Table 1:** Raman mode frequencies computed for reaction products and selected intermediates from reaction schemes in Fig. 4

**Products**		
Vibrational mode	VI (cm^−1^)	IX (cm^−1^)
CH_2_ wag	**1025**	**1023, 1216**
Ring stretch	**1125, 1147**	**1126, 1151**
CH_2_ wag and ring stretch	**1170**	**1171**
Ring stretch	**1279**	**1278**
CH_2_ scissor	1517, 1539	1536, 1553
C–H stretch	3116, 3147, 3231	3155, 3162, 3254
H_2_O related	NA	1654, 3373, 3749
**Graphene related**	1344, 1586, 1662	1363, 1584, 1652

**Intermediates**			
Vibrational mode	IV (cm^−1)^	V (cm^−1^)	VII (cm^−1^)
C–C/C–O stretch	1106	**1040**	897, **1056**
CH_2_ wag	**1157**	**1133, 1165**	1093, 1326
CH_2_ wag	1306, 1401	**1264, 1286**	**1151**, 1204
CH_2_ scissor	**1475**	**1415**	**1489**
C–H stretch	3023, 3160, 3295	3030, 3058, 3103	3065, 3176, 3333
**Graphene related**	1340, 1576, 1653	1349, 1580, 1657	1365, 1581, 1664

Those modes observed in in situ SERS studies are shown in bold

The presence of surface graphenic carbon appears to be a necessary condition for the observation of these reaction species in SERS spectra. None of the emitters that lacked graphenic carbon bands showed any vibrational modes from reaction species at any time in their SERS monitoring. Furthermore, as demonstrated by digital time trajectories of individual emitters under photocatalytic conditions (Fig. 2a and other examples in Supplementary Fig. 7), reaction species appear stochastically in the course of SERS monitoring, but the species are observed only (with few exceptions, if any) when graphenic carbon is also present on the surface. Thus, the active form of the photocatalyst appears to be a Ag NP surface covered with graphenic carbon fragments generated in situ under laser excitation.

To further verify the role of the graphene, we performed a SERS study of a catalyst consisting of well-dispersed, SiO_2_-supported Ag NPs, which were pre-covered by pristine graphene flakes. The reaction conditions (C_2_H_4_ + ambient air + 514.5 nm excitation) were similar to the case of the Ag NP catalyst without pre-covered graphene. As seen from the time series of SERS spectra (Fig. 2b), well-defined D and G bands from the graphene covering are present even prior to C_2_H_4_ introduction and stay persistent over the entire time course of the monitoring. Upon the introduction of C_2_H_4_, sharp peaks at 1145, 1270, 1415, and 1475 cm^−1^ corresponding to the epoxidation product (EO) or related reaction species^29,30^ (see Table 1 and Supplementary Table 1) appear stochastically, as shown by select SERS spectral frames (Fig. 2c).

We further examined this close association between the presence of graphene on the Ag surface and observation of photo-oxidation reaction species. Despite suggestions, additional amplification of Raman scattering by graphenic carbon has been ruled out by recent careful studies^31^. Graphenic carbon can, however, enrich molecular species at the surface by providing adsorption sites, which could aid the observation of these species by SERS, as demonstrated in our DFT calculations (Supplementary Figs. 8–12)^32–35^. However, the role of graphenic carbon goes above and beyond such an enrichment/enhanced detection effect. In fact, graphenic carbon actively promotes the C_2_H_4_ photo-epoxidation reaction on the Ag NP surface, which is confirmed in our bulk photocatalysis studies.

### Graphene/Ag/Al_2_O_3_ catalyst for C_2_H_4_ photo-epoxidation

**Fig. 3:**
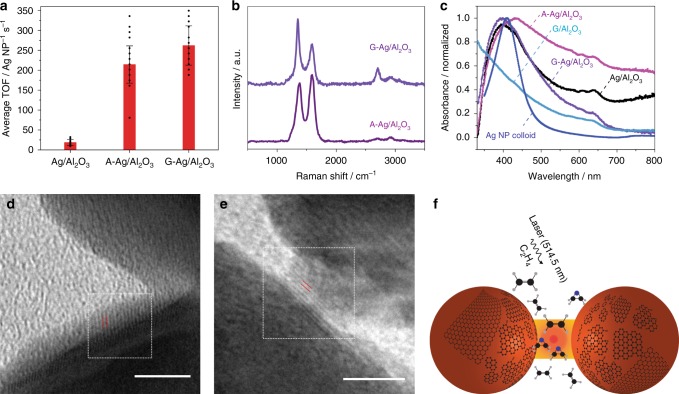
Graphene/Ag composite photocatalysts. **a** The C_2_H_4_ epoxidation activity, measured in terms of the turnover frequency (TOF) of EO generation, in a quasi-steady-state photocatalytic measurement for three different photocatalysts: Ag/Al_2_O_3_, G-Ag/Al_2_O_3_, and A-Ag/Al_2_O_3_. In each case, the bar height represents the average TOF from multiple cycles comprising a long photocatalytic reaction (see Supplementary Fig. 15) and the error bar represents the standard deviation of the TOF. Dot plots of the TOF for each cycle are also shown overlaid with the bar graph. **b** Normalized SERS spectrum of A-Ag/Al_2_O_3_ (after 14 h of irradiation) in air shows D and G bands, similar to G-Ag/Al_2_O_3_ indicating the formation of graphenic carbon on the Ag NP surface after activation. **c** Ultraviolet–visible extinction spectrum of Ag NP colloid in water and diffuse-reflectance absorption spectra of Ag/Al_2_O_3,_ A-Ag/Al_2_O_3_ (after 14 h of irradiation), G/Al_2_O_3_, and G-Ag/Al_2_O_3_ powders. The spectra are shown normalized; representative HRTEM images (scale bars = 5 nm) of Ag NPs irradiated by 514.5 nm laser (5 mW, ×60) in C_2_H_4_ showing **d** multi-layer graphene with interlayer spacing of 0.34 nm and **e** the {002} planes of graphene with interplanar spacing of 0.32 nm. Lattice fringe spacings were determined from FFT of the selected regions indicated by the square. **f** A schematic showing the formation of graphenic carbon on Ag NP surface under photocatalytic reaction conditions

The insight about the involvement of graphenic carbon in the active form of the photocatalyst has utility for practical catalyst design. As a demonstration, we prepared a composite catalyst consisting of graphene-functionalized Ag NPs supported on α-Al_2_O_3_ (G-Ag/Al_2_O_3_). We compared the photocatalytic C_2_H_4_ epoxidation performance (Fig. 3a and Supplementary Figs. 13–15) of this composite to the more conventional form of the catalyst consisting of Ag NPs supported on α-Al_2_O_3_ (Ag/Al_2_O_3_). The Ag loading (~20 wt%) was identical in the two cases. Both catalysts (G-Ag/Al_2_O_3_ and Ag/Al_2_O_3_) have an absorbance band spanning from ca. 400 nm extending out across the visible region of the spectrum (Fig. 3c). This visible absorbance is the contribution from the plasmonic absorption of Ag NPs and coupled NP aggregates, as ascertained from the lack of such absorbance in G-Al_2_O_3_ powder (Fig. 3c).

The reaction was carried out in a glass reactor filled with 15 mg of the solid catalyst powder, C_2_H_4_, and ambient air under continuous-wave excitation by a 0.5 W, 514.5 nm laser. As shown in Supplementary Fig. 15, G-Ag/Al_2_O_3_ showed relatively high and sustained activity towards photocatalytic epoxidation, as determined from EO generated in the reactor headspace measured by gas chromatography (GC). The average TOF for EO formation (Supplementary Note 1) was found to be 262 NP^−1^ s^−1^ (Fig. 3a) under the visible-light photoexcitation conditions employed. While this epoxidation activity is not comparable to that of thermal C_2_H_4_ epoxidation under industrial conditions, the accomplishment of epoxidation under ambient conditions is noteworthy. The quantum yield of the photoreaction is estimated to be 5.75 × 10^−5^ EO molecules generated per photon (Supplementary Note 2).

A control test in which G-Ag/Al_2_O_3_ was excited by the 514.5 nm laser in air atmosphere in the absence of C_2_H_4_ yielded no EO (Supplementary Fig. 13), confirming that EO was an epoxidation product and not an outcome of graphene photodegradation. G-Al_2_O_3_ itself exhibited no activity (Supplementary Fig. 13), confirming that the Ag NPs are required as a light absorber in the photocatalytic reaction. In contrast to the high activity of the composite catalyst (G-Ag/Al_2_O_3_), as-prepared Ag/Al_2_O_3_ showed low activity for photo-epoxidation (Fig. 3a and Supplementary Figs. 14, 15), despite its significant light absorption at 514.5 nm (Fig. 3c). Thus, we confirmed that for visible-light-driven C_2_H_4_ epoxidation at ambient conditions, the photocatalyst requires both graphene and Ag NPs. Interestingly, we found that the as-prepared Ag/Al_2_O_3_ could be activated under visible-light irradiation in C_2_H_4_ atmosphere. The activated catalyst (A-Ag/Al_2_O_3_) obtained from 14 h of such irradiation exhibited a photo-epoxidation performance much higher than Ag/Al_2_O_3_ and one approaching that of G-Ag/Al_2_O_3_ (Fig. 3a and Supplementary Fig. 15). Naturally, we conjectured that the activation process is tantamount to the in situ formation of graphenic carbon on the surfaces of Ag NPs. The latter was indeed the case as seen from SERS spectra of A-Ag/Al_2_O_3_ showing distinct D and G bands (Fig. 3b).

HRTEM imaging (Fig. 3d, e and Supplementary Fig. 16) provided additional characterization of the in situ formed graphenic carbon/Ag NP composite. The as-received Ag NPs are quasi-spherical in shape. Their crystallinity is evident from lattice spacings of 0.24 and 0.21 nm, corresponding to {111} and {200} planes of metallic Ag (Supplementary Fig. 16a–c)^36–39^. Occasionally, we observe a lattice spacing of 0.28 nm corresponding to the {111} planes of Ag_2_O^38–40^, possibly formed by exposure of the Ag NPs to ambient air prior to TEM analysis. HRTEM images of Ag NPs irradiated with 514.5 nm laser in ambient air (Supplementary Fig. 16d–f) indicate no obvious structural or morphological changes resulting from solely laser irradiation. However, Ag NPs subjected to 514.5 nm laser irradiation in C_2_H_4_ (similar to in situ SERS and photocatalytic reaction conditions) show on their surface the presence of graphenic carbon nanofragments, with an interlayer spacing of 0.34 nm (Fig. 3d). This signature interlayer spacing of 0.34 nm is confirmed from HRTEM images of Ag NPs covered with commercially available graphene flakes (Supplementary Fig. 16g–i). The {002} planes of graphene with a lattice spacing of 0.32 nm^41–44^ are also seen in HRTEM of the graphenic carbon fragments formed on Ag NPs by irradiation in C_2_H_4_ (Fig. 3e). Defective or partially oxidized graphene^45,46^ with an interlayer spacing of 0.54 nm is also observed in HRTEM (Supplementary Fig. 17).

These bulk photocatalytic and HRTEM studies verify that the in situ formation of graphenic carbon (depicted in Fig. 3f) is a primary step of the photocatalytic reaction, and the graphene/metal composite acts as the active phase of the photocatalyst under reaction conditions. Graphenic carbon catalytically promotes C_2_H_4_ photo-epoxidation, which is in striking contrast to the accepted role of carbon as an innocent support or a deactivator in conventional catalysis. Although these mechanistic findings may not apply to the industrial epoxidation reaction, which is conducted at significantly elevated temperatures and pressures, the formation of graphene and its role as a catalytic promoter may be relevant to other photocatalytic hydrocarbon transformations performed under ambient conditions.

### DFT insights into role of graphene in catalytic promotion

**Fig. 4:**
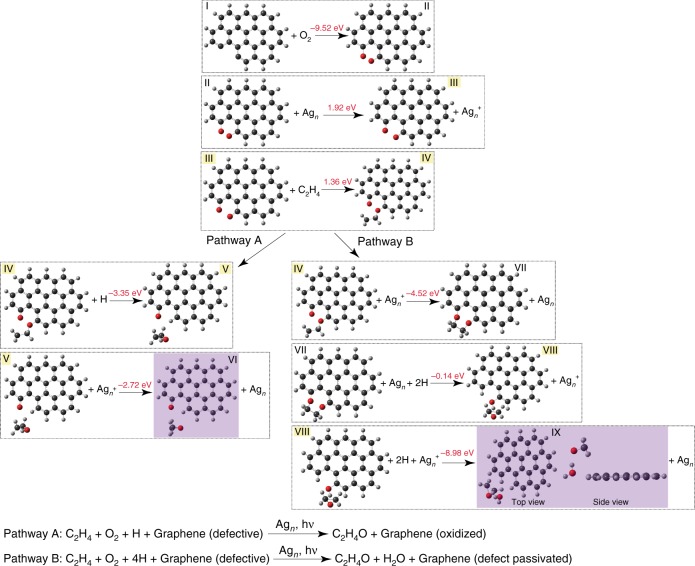
DFT simulation of the role of graphene nanofragments in catalysis. Two possible reaction pathways by which unterminated bonds of graphene nanofragments can participate in C_2_H_4_ epoxidation. Here and in molecular geometries in the Supplementary Information, O atoms are shown in red, C atoms in dark gray, and H atoms in light gray. Ag_*n*_ refers to the 15-atom Ag cluster model employed in DFT simulations. The arrow within each step signifies that a geometry optimization of the chemical species on the left leads to the species on the right. The product of each reaction pathway is shown by purple shading. Free energies for each elementary step are shown (atop each arrow), which were calculated from optimized geometries of gas phase species (I–IX) involved in each step. The species highlighted in yellow were assigned a −1 charge, to simulate the injection of a hot electron from Ag. The overall reaction for each pathway is shown at the bottom

The possible mechanism/s by which graphene nanofragments promote C_2_H_4_ epoxidation was investigated using DFT simulations. We focused our attention on unterminated bonds of the graphene nanofragments, which are likely highly active sites for chemisorption. Note that pristine graphene flakes can form such active nanofragments under laser excitation, with assistance from the light-absorbing Ag NP (Supplementary Figs. 4, 6). DFT computations of free energies show that both C_2_H_4_ and O_2_ can be chemisorbed at unterminated bonds of a graphene nanofragment with high energetic favorability (Fig. 4 and Supplementary Figs. 8 and 9). Such chemisorption can be a first step in C_2_H_4_ epoxidation.

Figure 4 shows a possible pathway starting from O_2_ chemisorption at an edge defect lacking H termination (I → II). Following O_2_ incorporation, a photogenerated hot electron transfers from Ag_*n*_ (II → III) can trigger the scission of the O–O bond. The 1.92 eV cost of this dissociation reaction can be compensated by the excess energy (relative to the Ag Fermi level) of the hot electron. One of the dangling O atoms can further react with a C_2_H_4_ molecule from the gas phase, while the other O atom is involved in a structural reorganization to a ketone. The C=O vibrational mode (1720–1740 cm^−1^) observed in in situ SERS (Supplementary Fig. 5j) may be a signifier of such ketone formation. This step (III → IV) requires a free energy of 1.36 eV, posing an activation barrier for the reaction.

The anion intermediate (IV) can release EO by one of two possible reaction pathways. Reaction of the anion intermediate with the hole leftover in the Ag_*n*_ and an H˙ atom generates an EO molecule and a ketone and H termination at the defect (Fig. 4, Pathway A). Alternatively, the anion can react with the hole leftover in the Ag_*n*_ and four H˙ atoms to result in an EO molecule, a water molecule, and H termination at the defect (Fig. 4, Pathway B). Ag NP-photocatalyzed scission of C–H bonds in C_2_H_4_ (during the condensation reaction) or in graphene can serve as the source of H˙ atoms, which can stay adsorbed to the Ag surface. Although we present two of possibly many pathways, they highlight the ability of graphene nanofragments to facilitate the C_2_H_4_ photo-epoxidation reaction in synergy with photogenerated carriers available from the plasmonically excited Ag NP.

The addition of reactant species (C_2_H_4_ or O_2_) to high energy defects of a graphene fragment can effectively passivate them, resulting in the termination of their participation in catalysis. Yet, sustained photo-epoxidation activity is exhibited by graphene-containing photocatalysts (G-Ag/Al_2_O_3_ and A-Ag/Al_2_O_3_), with no observed deactivation (Supplementary Fig. 15), which points to the regenerability of catalytic graphene sites. Visible-light activation can promote continuous regeneration of active graphene defect sites through multiple photo-mediated scission reactions and/or the photo-assisted condensation of new graphene nanofragments with unsaturated edges. Such a proposal is supported by the dynamic formation and breakdown of graphenic carbon fragments observed in SERS monitoring (Fig. 2a and Supplementary Figs. 4, 6).

We computed Raman spectra of the reaction intermediates and products of the two reaction pathways. As shown by the calculated Raman frequencies (Table 1) and spectra (Supplementary Fig. 11), in both pathways, the formation of EO contributes modes at 1023, 1145, 1170, 1216, and 1277 cm^−1^, which reproduce the SERS bands observed experimentally (Figs. 1h, 2c and Supplementary Fig. 5). Adsorption free energy calculations (Supplementary Table S2) suggest that EO generated at a defect site can adsorb to the Ag NP surface (∆*G*_ads_ = −0.53 eV) for a timescale long enough to be detected by SERS. Scissoring modes of CH_2_ in the reaction intermediates contribute to computed vibrational modes at 1415 and 1470 cm^−1^, which also correspond to bands in experimental SERS spectra (Figs. 1h, 2c and Supplementary Fig. 5). Note that vibrational modes in a similar range (1432 and 1484 cm^−1^) have been reported in the literature and were assigned to a oxametallacycle intermediate formed on the Ag surface in thermal C_2_H_4_ epoxidation^29^.

In conclusion, using SERS performed in an ensemble-averaging-free manner, we captured and identified the dynamic formation of graphenic carbon fragments on the surface of an Ag catalyst, which constitutes a primary step in photocatalytic C_2_H_4_ epoxidation. The composite of this in situ formed graphenic carbon with the plasmonic Ag NP constitutes the catalytically active phase of the photocatalyst. This atomic-level insight coupled with DFT simulations sheds light into an unconventional role of crystalline carbon as a promoter in the photocatalysis and also allows us to engineer an improved photocatalyst for ambient-condition C_2_H_4_ epoxidation. The findings highlight the central importance of in situ studies of photocatalysts, where the pristine metal surface may not solely be the active phase of the photocatalyst under reaction conditions.

## Methods

### Flow cell preparation

An optical microscopy-compatible flow cell was constructed from a glass slide and a glass coverslip. Glass coverslips (VWR SuperSlips, No.1, 24 × 60 mm^2^) and glass slides (VWR, 1 mm thick, 75 × 25 mm^2^) were cleaned by etching away the top silica surface by heating in 2 M KOH for 30 min (~ 85 °C), followed by washing first with distilled water, and then with Nanopure water. The glass slides and coverslips were sonicated in Nanopure water for 15 min. Further again, the coverslip was washed with Nanopure water and sonicated again in fresh Nanopure water for 30 min. A 1 mm (diameter) and a 2.5 mm hole were drilled at a distance of 1 cm, respectively, from the left and right edges of a glass slide using a diamond-coated drill bit operated at ~15,000 rpm, immersed in water.

The sample was prepared from PELCO^®^ NanoXact^TM^ citrate-capped Ag NP colloid (Ted Pella, Item no. 84050-60), unless otherwise specified. TEM images of these colloids (Supplementary Figs. 1a–c) show quasi-spherical NPs of average size 52.5 ± 5.5 nm and consisting of metallic Ag. Ten microliters of this colloid, which has a localized surface plasmon resonance (LSPR) absorption band maximum at ~432 nm (see Supplementary Fig. 2), were diluted with 40 µL of Nanopure water. The mixture was sonicated for a few minutes. The precleaned coverslip was blow dried with N_2_ gas. Then, 10 µL of the diluted NP dispersion was drop-casted onto the coverslip and allowed to dry at room temperature to obtain well-segregated NP emitters. The coverslip-supported sample was then subjected to a cleaning method for removing citrate ligands from the surfaces of the NPs. The sample-coated coverslip was heated in a vacuum desiccator at 80–90 °C for 5 h (including time for ramping up to high temperature) and then allowed to cool. After such pretreatment, a large fraction of the Ag NP emitters was found to be free of background SERS signal from citrate. The coverslip was incorporated into the flow cell. Briefly, a precleaned glass slide was blow dried with N_2_ gas. A polyethylene tube (Instech laboratories, 0.076 cm inner diameter) was inserted into the 1 mm hole (the inlet of the flow cell) and glued using 5 min Loctite epoxy. The epoxy was allowed to polymerize for 20 min after mixing. The 2.5 mm hole was left unsealed to serve as the outlet for the flow cell. Epoxy glue was applied on the inner edge of glass slide, to which the NP-coated coverslip was attached. Tens of µm-thick double-sided tape served as a spacer between the coverslip and the glass slide.

### Ag NP monolayer preparation

Some SERS experiments were carried out on Ag NP monolayers (Fig. 1j). In a typical procedure, a precleaned coverslip was immersed in a 1% (v v^−1^) solution of 3-aminopropyltriethyoxysilane in ethanol for 1 h. Following this aminosilanization procedure, the coverslip was thoroughly washed with ethanol, then with Nanopure water, followed by drying with N_2_. A successfully prepared aminosilanized coverslip exhibits strong hydrophobicity, which is clearly observed by water beading up on the coverslip surface. The coverslip was then placed in a Ag NP dispersion for at least 16 h to allow the amine-terminated surface to be coated with a monolayer of Ag NPs. The initially transparent coverslip turned greenish following the coating of Ag NPs. To remove citrate ligands from the surfaces of the NPs and remaining aminosilane, the coverslip was washed with Nanopure water followed by drying under under N_2_ and then placed for 30 min in a freshly prepared 50 mM aqueous solution of NaBH_4_, which was followed by thorough washing with Nanopure water^47^. This NaBH_4_ treatment was repeated three times. For ensuring more complete cleaning, the sample-coated coverslip was irradiated with ultraviolet (UV) light from a mercury lamp (46 W) for photooxidative removal of citrate ligands. The duration of the pretreatment varied from one sample to another, but generally a 10 min exposure was sufficient to yield Ag NP emitters with no SERS backgrounds. Note that this method can cause some oxidation of the Ag NP surface; however, we found the SERS response to be qualitatively consistent with samples cleaned by vacuum baking. The monolayer-coated coverslip was then assembled into a flow cell for in situ SERS measurements.

### Graphene-covered Ag NPs for in situ SERS measurement

First, 10 μL of Ag NP colloid, diluted 5× with Nanopure water, was drop-casted onto a precleaned coverslip and subjected to a ligand cleaning procedure described above. A 10 μL ethanolic solution of graphene in (0.1 mg mL^−1^, Electron Microscopy Sciences) was then drop cast onto the Ag NP-coated coverslip and dried by heating at 60 °C in a vacuum desiccator for 2 h. The sample-bearing coverslip was then assembled into a flow cell. The in situ SERS measurements on the graphene-covered Ag NP sample were carried out in a manner similar to that for the Ag NP sample.

### Dark-field scattering imaging of Ag NP emitters

The flow cell was mounted in an inverted Olympus microscope with the coverslip side facing down towards the microscope objective. The Ag NP-coated surface was illuminated with an Olympus U-LH100-3 100 W halogen lamp focused through an Olympus U-DCW 1.2–1.4 NA oil immersion dark-field condenser. Light scattered from the Ag NPs was collected in dark-field mode using an Olympus UPlanApo 0.5–1.35 NA ×100 oil immersion objective. For dark-field imaging, the focus of the condenser and that of the objective was set to the surface bearing the Ag NPs. Ag NP emitters were visualized in the wide-field image due to their strong LSPR scattering. Selected emitters were aligned with a spectrometer slit mounted on the exit port of the microscope for in situ SERS spectroscopy. For the Ag NP monolayer, similar dark-field imaging was carried out. The colored dark-field images (Fig. 1i and Supplementary Fig. 3c, d) were taken using a top-view camera (AmScope MU035) mounted on the microscope and a ×100 dark-field objective.

### In situ SERS studies

The flow cell was mounted in an inverted Olympus microscope with the coverslip side facing down towards the microscope objective. The Ag NP-coated surface of the flow cell was excited by an Ar laser beam (Stabilite 2017, 514.5 nm, 5 mW) focused to a spot (full-width half-maximum (fwhm) = 2.56 µm)^47^ using an Olympus UPlanApo ×60 water immersion objective. The backscattered light was collected by the same objective and detected in the form of a spectrum using a Princeton Instruments Acton SP-2358 spectrograph equipped with a 300 lines per mm, 500 nm blaze grating and a Pylon 100B charge-coupled device (CCD) camera. A selected Ag NP emitter identified in the wide field was aligned with the spectrometer slit mounted on the exit port of the microscope. Prior to the introduction of C_2_H_4_, SERS spectra were continuously recorded for the selected emitter for hundreds of frames with an acquisition time of 0.2 s per frame and the spectrograph centered at 660 nm. The absence of any background SERS bands, say from ligands, was ensured prior to further study of the selected emitter. Following this control run, pure C_2_H_4_ was allowed to flow out from a balloon into the flow cell inlet and allowed to exit through the open outlet. Air, which serves as the source of oxygen, was not excluded from the flow cell. Typically, the introduction of the gas causes a pressure rise inside the flow cell, leading the cell to slightly buckle and move the Ag NP emitter out of focus. The objective lens had to be slightly readjusted to regain focus. Under C_2_H_4_ flow, SERS spectra were recorded continuously with an acquisition time of 0.2 s per frame with the spectrograph centered at 660 nm. The start of the experiment is set as *t* = 0 s in plotted time series. Typically, after acquiring 1000–2000 frames of spectra, the C_2_H_4_ flow was turned off. Another suitable emitter was identified and aligned with the spectrograph slit and subject to a similar control run, followed by in situ SERS spectroscopy in C_2_H_4_.

### Data analysis

Hand in hand with the single-molecule-level sensitivity of SERS, spectra acquired with single NP spatial resolution and sub-second time resolution naturally reflect spatial and temporal heterogeneities present in the Ag NP photocatalyst sample (Supplementary Fig. 5). Such heterogeneities are an inherent feature of heterogeneous surfaces. Unlike ensemble-averaged or less sensitive techniques, SERS manages to capture these inherent site-to-site variations. Nevertheless, in the presence of such variabilities, for making meaningful findings, we relied on a comprehensive analysis of the large catalog of 1000–2000 of frames of SERS spectra acquired from 220 single NPs across several samples studied. Only observations that were well represented in this large catalog of spectra were considered to be statistically meaningful.

The SERS spectra recorded in Winspec were analyzed using Matlab or Origin software. Water-fall plots were made from time stacks of spectra, with each spectrum normalized from 0 to 1. In specific cases, a single-frame SERS spectrum is selected from the continuous time series for analyzing representative behavior. In these cases, the spectrum was first subject to manual baseline subtraction in Origin and plotted with or without normalization from 0 to 1. SERS peaks were identified manually and labeled with their wavenumber maxima. SERS peaks were assigned manually to known vibrational modes tabulated in Table S1 and Table 1.

For some representative cases, digital trajectories were generated for each of the key reaction species: EO, CO_2_, and graphene. A time series of SERS spectra was analyzed frame by frame by an automated Matlab code. Spectra were first smoothed and peaks were located by a first-derivative method. If a spectrum contained three or more vibrational modes corresponding to EO (1025, 1147, 1170, 1279, 1415, 1475, and 1530 cm^−1^ with a ±20 cm^−1^ tolerance), then EO was assigned to be present in that frame. CO_2_ was assigned to be present if both signature modes (1283 and 1380 cm^−1^ with a ±20 cm^−1^ tolerance) were found in a spectrum. Graphenic carbon was assigned to be present if either 1330 and 1560 cm^−1^ bands (with a ±30 cm^−1^ tolerance) were present. Spectral frames with SERS modes from the specific reaction species were assigned a value of 1. Frames where modes from the species were absent were assigned a value of 0. The presence of the species (assigned a value of 0 or 1) was plotted as a function of time, with *t* = 0 s set to the start of the experiment.

### DFT simulations

DFT calculations were performed using Gaussian 09 installed on a supercomputing cluster. Input geometries of reaction species (gas phase, unless noted otherwise) and model Ag clusters were created in Gauss View or Avogadro softwares. According to our TEM study (Supplementary Fig. 16), Ag (111) is the most prevalent surface facet on the Ag NPs. Therefore, the Ag surface was modeled by the (111) surface of a 2 × 2 × 3 slab of Ag, consisting of a total of 15 atoms^29^. The Ag_2_O and AgO model clusters were generated using VESTA (version 3.3.2) software using reference crystal structure information from Springer Materials (http://materials.springer.com/). Crystal structures were exported from VESTA in the form of xyz files, which were converted into Gaussian input files using Avogadro. All other input geometries were built using Gauss View.

Geometries of reactive species were optimized in Gaussian 09. Model Ag clusters were optimized separately and free energies were determined. Ag atoms were described by LANL2DZ basis set^48^, which treats the outer 19 electrons of Ag atom with a double zeta basis set and treats all the remainder electrons with the effective core potential of Hay and Wadt^49^. All non-metallic atoms (C, H, and O) were described by the 6-31G** basis set of double zeta quality. All calculations were spin unrestricted and used the B3LYP hybrid functional proposed by Becke et al.^50^, which includes a mixture of Hartree–Fock and DFT exchange terms associated with the gradient-corrected correlation functional of Lee et al.^51^ Ag atoms were allowed to relax until the force on all atoms converged to 0.05 eV Å^−1^. For calculations on reaction species without the Ag surface, the geometry was relaxed until the force on all atoms converged to the default value of 0.008 eV Å^−1^. Raman mode frequencies and activities were calculated for the optimized geometries. Note that computed Raman mode activities do not reproduce SERS intensities, because the latter depend on the local field enhancement factors for the vibrational modes in question; only mode frequencies can be reproduced by calculations. Thermodynamic parameters were extracted from the vibrational mode calculation at 298 K and 1 atm, which are default settings. The free energy of a reaction was calculated as:

1 $$\Delta _{\mathrm{r}}G = \sum \left( {\varepsilon _{\mathrm{o}} + G_{\mathrm{corr}}} \right)_{{\mathrm{products}}} - \sum \left( {\varepsilon _{\mathrm{o}} + G_{\mathrm{corr}}} \right)_{{\mathrm{reactants}}},$$

where (*ε*_o_ + *G*_corr_) represents the sum of electronic and corrected thermal free energies. Optimized geometries were plotted in Gauss View or Avogadro. Details of energy calculations are provided in the Supplementary Notes.

### Synthesis of Ag/Al_2_O_3_

Citrate-capped Ag NPs were synthesized using the Lee and Meisel method^52^. Briefly, 45 mg of AgNO_3_ was dissolved in 250 mL of Nanopure water and the solution was brought to boiling. Five milliters of 1 wt% aqueous sodium citrate solution was then injected and the solution was kept boiling for 1 h. The brownish as-synthesized Ag NP colloid was centrifuged at 6300 × *g*, followed by washing with Nanopure water 3X and re-dispersed in ~30 mL of Nanopure water. The LSPR maximum of this colloid was found from UV–Vis absorbance spectroscopy to be at ~409 nm (Fig. S2).

Assuming 100% of the AgNO_3_ precursor in the synthesis was converted into Ag NPs, the colloid contained ~25 mg of Ag by weight. To this suspension, 100 mg of α-Al_2_O_3_ was added, followed by sonication for 1 h. The Ag:Al_2_O_3_ is in a 1:4 ratio by weight. The Ag/Al_2_O_3_ mixture was heated at a 60 °C under stirring until all the water was evaporated. The powder left behind was recovered and dried in a vacuum desiccator overnight under heating at 60 °C.

### Synthesis of G/Al_2_O_3_

Fifty-six milligrams of Al_2_O_3_ powder was dispersed in 20 mL of ethanol under sonication. Then, a 280 μL solution of graphene in ethanol was added to the dispersion, followed by sonication for 20 min. The solution was heated at 50 °C under stirring until the ethanol fully evaporated. The powder left behind was recovered and dried in vacuum desiccator overnight under heating at 60 °C.

### Synthesis of G-Ag/Al_2_O_3_

Seventy milligrams of Ag/Al_2_O_3_ powder, synthesized as above, was dispersed in 20 mL of ethanol under sonication. Then, a 280 μL solution of graphene in ethanol was added to the dispersion, followed by sonication for 20 min. The solution was heated at 50 °C under stirring until the ethanol fully evaporated. The power left behind was recovered and dried in vacuum desiccator overnight under heating at 60 °C.

### Activation of Ag/Al_2_O_3_

The Ag/Al_2_O_3_ powder was placed in a glass vial, which was then flushed with pure C_2_H_4_ for 30 min. The powder was then irradiated by a 514.5 nm laser (1 W, 1 cm beam diameter) for 14 h. This activation process, the product of which was referred to as A-Ag/Al_2_O_3_, was carried out prior to a photocatalytic reaction.

For each powder sample, a diffuse-reflectance absorption spectrum was obtained. Two kinks in the spectra resulting from detector/light source change at 348 and 800 nm were removed, followed by normalization of the spectra from 0 to 1, prior to plotting.

### Bulk photocatalysis studies

Photocatalytic reaction studies for G/Al_2_O_3_, Ag/Al_2_O_3_, G-Ag/Al_2_O_3_, and A-Ag/Al_2_O_3_ powders were carried out in batch mode in a glass tube reactor (9 mL volume), with the light-absorbing catalyst lying at the bottom of the tube. The reactor was sealed by a rubber septum fastened by a copper wire. Ambient air was used as the source of molecular oxygen (O_2_). Prior to reaction, the reactor containing the catalyst was flushed with house air for 30 min, after which the reactor contains 9 cm^3^ of air, equivalent to ca. 1.89 cm^3^ of O_2_. Then, 2 cm^3^ of C_2_H_4_ at atmospheric pressure was injected into the reactor and mixed well using a syringe. The catalyst was irradiated with a laser (514.5 nm, 0.5 W, 2 mm beam diameter) to initiate the photocatalytic reaction. The amount of catalyst (15 mg) loaded into the reactor was more than sufficient to cover the laser beam area, in order to ensure complete light absorption. The reaction was paused at 2.5-min intervals by blocking the laser and reaction products in the reactor headspace were analyzed by GC. An Agilent 6850 GC with an HP-Plot Q capillary column equipped with a flame ionization detector (FID) was used to assay the reaction mixture. The GC column was held at 50 °C for 4.5 min, then raised to 60 °C at the rate of 20 °C min^−1^, where it was maintained for 3 min, followed by ramping of the temperature to 100 °C at the rate of 20 °C min^−1^, where it was maintained for 5 min. Finally, the oven temperature was ramped to 160 °C at a rate of 20 °C min^−1^ and held for 10 min. Helium was used as the carrier gas at a flow rate of 1 mL min^−1^. The injector and the detector temperatures were set at 150 and 200 °C, respectively. C_2_H_4_ and EO peaks were identified based on retention times, which were measured to be around 3.4 and 13.9 min, respectively, from GC of C_2_H_4_ and EO standards. The peak area corresponding to EO was integrated in GC Chemstation software. We ran 3–4 trials for each catalyst to account for batch-to-batch variability in activity. We also ran two trials of control photoreactions (in the absence of C_2_H_4_ but with 9 cm^3^ air) for Ag/Al_2_O_3_ and G-Ag/Al_2_O_3_. We also ran two trials of a control photoreaction for G/Al_2_O_3_ in a reactor filled with 9 cm^3^ air and 2 cm^3^ C_2_H_4_.

The EO for the standard run was synthesized using a wet chemistry method^53^, whereby a mixture of 3 mL of 14.9 M 2-chloroethanol (Sigma Aldrich, USA) and 7.5 mL of 6 M aq. KOH solution (Fisher Scientific, USA) was gently heated in an oil bath at ~80 °C. The EO gas generated in the reaction (Cl-CH_2_CH_2_-OH + KOH → (CH_2_CH_2_)O (g) + KCl + H_2_O) was collected by a balloon. Standard GC runs were also conducted for a hydrocarbon mixture and acetaldehyde.

Photocatalytic reaction studies for Ag/Al_2_O_3_, G-Ag/Al_2_O_3_, and A-Ag/Al_2_O_3_ were also performed under quasi-state conditions by adapting the above procedure as follows. First, 15 mg of the catalyst powder was loaded into a glass reactor. The reactor was sealed and flushed with house air (10  sccm) for 20 min, after which the reactor contains 9 cm^3^ of air. Then 2 cm^3^ of pure C_2_H_4_ gas was injected into the reactor and mixed well using a syringe. The catalyst powder was irradiated in the C_2_H_4_ and air atmosphere by a 0.5 W, 514.5 nm laser. After 2 min of photoreaction, 100 μL of the headspace vapor of the reactor was sampled and subject to GC-FID analysis. After this, the reactor was opened and evacuated by subjecting it to vacuum for 10 min at room temperature. After evacuation, the reactor was sealed and filled with air and C_2_H_4_, using the aforementioned procedure. The catalyst powder in the gas atmosphere was then subject to a 2-min photoreaction and GC-FID analysis. A 90-min (cumulative) long photocatalytic reaction was conducted in such a cyclic manner, the results of which are presented in Supplementary Fig. 15.

The amount of EO generated in each cycle was quantified using the integrated area measured for the EO peak (14 min retention time) in the gas chromatogram. For this quantification, a calibration was separately carried out using pure EO gas of a known concentration (100 ppm in N_2_, Cal Gas Direct, Inc.). Estimation of TOF of EO generation is described in the Supplementary Notes.

### SERS spectra of G-Ag/Al_2_O_3_ and A-Ag/Al_2_O_3_

G-Ag/Al_2_O_3_ or A-Ag/Al_2_O_3_ powder was dispersed in Nanopure water to a level of dilution where no clumping of the powder is seen. The dispersion was subsequently drop cast onto a precleaned coverslip, followed by drying with N_2_. The sample-bearing coverslip was mounted on the optical microscope and excited by a focused 514.5 nm laser (5 mW, ×60 objective, beam fwhm = 2.56 µm). Continuous SERS spectra were acquired in ambient air with 0.5 s per frame acquisition time. The method for acquiring SERS spectra was similar to that for the flow cell experiments.

### TEM sample preparation and imaging

Ten microliters of PELCO® NanoXact^TM^ 60 nm citrate-capped Ag NPs (Ted Pella, Item no. 84050-60) were sonicated for 5 min and drop-casted onto a TEM grid (ultrathin carbon film on a lacey carbon film supported by a 400 mesh Cu grid, Ted Pella). The solution was allowed to dry in ambient air for ~1 h and then heated in a vacuum desiccator at 80–90 °C for 5 h. In addition to the as-received Ag NPs, three other samples were subject to TEM:

(i) A sample of Ag NPs prepared on a TEM grid, as above, was attached using double-sided tape to a precleaned glass slide with sample facing away from the slide. The glass slide was integrated into a flow cell, which was mounted on the optical microscope. The sample was irradiated by a 514.5 nm laser (5 mW, ×60 objective, beam fwhm = 2.56 µm) in ambient air. A few sample-bearing grid squares at the center of the grid were subjected one at a time (by rastering the sample) to such irradiation for a time duration ranging from less than a second to tens of seconds.
(ii) A sample of Ag NPs prepared on a TEM grid was subjected to a similar procedure as (i), except the irradiation was carried out in an atmosphere of C_2_H_4_. Air was not excluded from the flow cell.
(iii) A sample of Ag NPs prepared on a TEM grid, as above, was then covered with commercially available graphene. Ten microliters of ethanolic solution of graphene (1 mg mL^−1^) was drop-casted onto the Ag NP-bearing grid and allowed to dry in air.

TEM and HRTEM imaging was performed on a Hitachi 9500 microscope operating at 200 kV with a spot size of 150 nm (diameter). In the case of (i) and (ii), imaging was conducted on the central region of the TEM grid, which had been laser-treated. Micrographs were acquired by an Orius CCD camera with an exposure time of 0.1 s and an acquisition time of 1 s. Micrographs were analyzed in ImageJ or Digital Micrograph software. Fast-Fourier transforms of selected regions were performed to determine lattice fringe distances. Scale bars were also included in ImageJ.

### Data availability

Data supporting the findings of this manuscript, Matlab codes, and computational input files are available from the corresponding authors upon reasonable request.

## Electronic supplementary material

Supplementary Information
