# Supplementary material for: In situ formation of catalytically active graphene in ethylene photo-epoxidation
Source: Nat Commun. 2018 Aug 3;9:3056. doi: 10.1038/s41467-018-05352-9 (PMC6076287; doi:10.1038/s41467-018-05352-9)
Supplement: Supplementary file 1 — Supplementary Information [file 41467_2018_5352_MOESM1_ESM.pdf]

Supplementary Information

In situ formation of catalytically active graphene in ethylene photo-epoxidation

Xueqiang Zhang,[1] Gayatri Kumari,[1] Jaeyoung Heo,[2] and Prashant K. Jain[1,3]*

[1]Department of Chemistry, University of Illinois at Urbana-Champaign, 600 S Mathews Ave, Urbana, IL 61801, USA

[2]Department of Materials Science and Engineering, University of Illinois at Urbana-Champaign, 1304 W Green St, Urbana, IL 61801, USA

[3]Materials Research Laboratory, University of Illinois at Urbana-Champaign, 104 S Goodwin Ave, Urbana, IL 61801, USA

*Corresponding author E-mail: jain@illinois.edu

Supplementary Tables

Supplementary Table 1. Compilation of known Raman vibrational modes from literature, partly based on which SERS peaks in our study were assigned.

Raman shift (cm^{-1})	Vibrations or species	Ref.
1740	C=O	1
1330, 1570	D and G band of graphene	2,3
1330, 1570, 2660, 2900, 3180	Graphene flakes (D, G, 2D, D+G, and 2G bands, respectively)	3
1360, 1610, 2720, 2970, 3180	Graphene oxide (D, G, 2D, D+G, and 2G bands, respectively)	2,4,5
1615–1625, 1342	Gas phase C_2H_4	1,6-8
1148, 1270 (1240–1280), 1411, 1477–1498, 3018	Ethylene oxide (EO) and oxametallacycle adsorbate	1,9
1283, 1380	Fermi resonance from $2v_2$, v_1 modes of CO_2	10
956–968	Stretching vibration of surface atomic oxygen Ag=O	11
866	Surface Ag-O-Ag	11
827	Ag-O/CH_2 rocking modes in C_2H_4	6,11
730	Peroxide	12,13
620	AgO, bulk	11
430	Ag_2O, bulk	14

Supplementary Table 2. Free energies of adsorption of reaction species on different surfaces in Supplementary Figure 12.

Surface Adsorbate	Adsorption energy / eV
Ag (111) + C_2H_4	0.69
Ag_2O + C_2H_4	0.68
AgO + C_2H_4	-1.04
Ag (111) + EO	-0.53
Graphene + C_2H_4	0.89
Graphene + EO	0.34
Graphene + O_2	1.70
Graphene + CO_2	0.21

Supplementary Figures

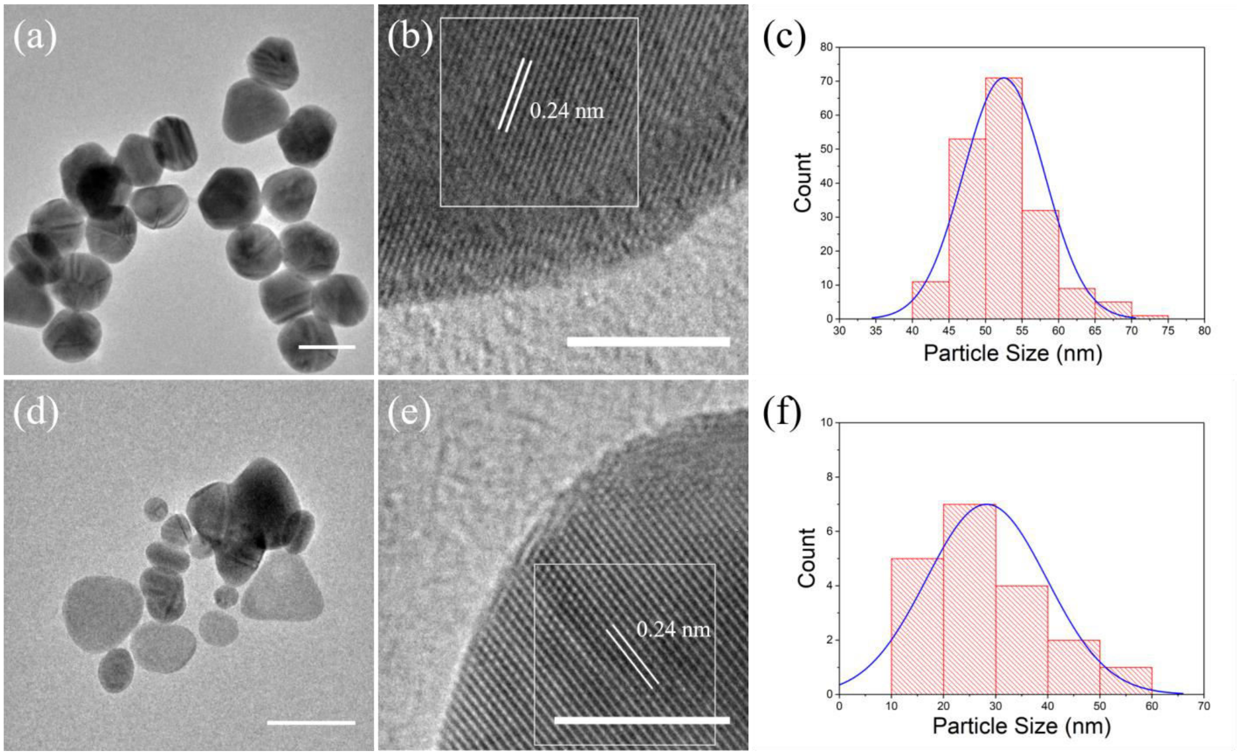

Supplementary Figure 1. Representative (a and d) transmission electron microscopy (TEM) images (with a scale bar of 50 nm) and (b and e) magnified high-resolution transmission electron microscopy (HRTEM) images (with a scale bar of 5 nm) of (a and b) citrate-capped Ag NPs purchased from Ted Pella and (d and e) polyethylenimine (PEI)-capped Ag NPs synthesized in the laboratory. HRTEM images show the crystallinity of the NPs and the presence of metallic Ag, as indicated by the lattice spacing of 0.24 nm corresponding to the {111} planes of Ag. (c and f) Size distribution histograms (along with a normal distribution fit shown by the blue curve) for (c) Ted Pella Ag NPs and (f) PEI-capped Ag NPs, which are found to have an average nanoparticle size (± standard deviation) of 52.5 nm (± 5.5 nm) and 28.3 nm (± 11.6 nm), respectively.

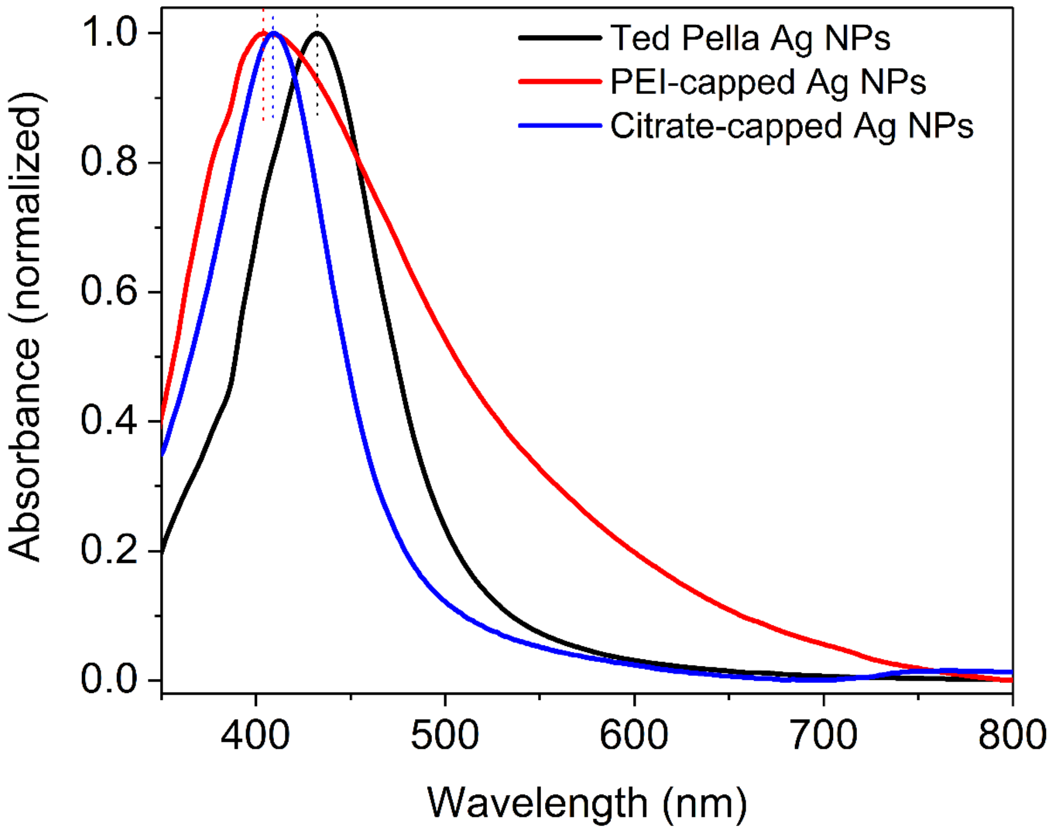

Supplementary Figure 2. UV–Vis extinction spectra (normalized) of the three types of Ag NPs employed in the study: Ag NPs purchased from Ted Pella (black line), polyethylenimine (PEI)-capped Ag NPs (red line) and citrate-capped Ag NPs (blue line). The LSPR maximum position, which is an indicator of NP size, is marked by a vertical dashed line for each spectrum.

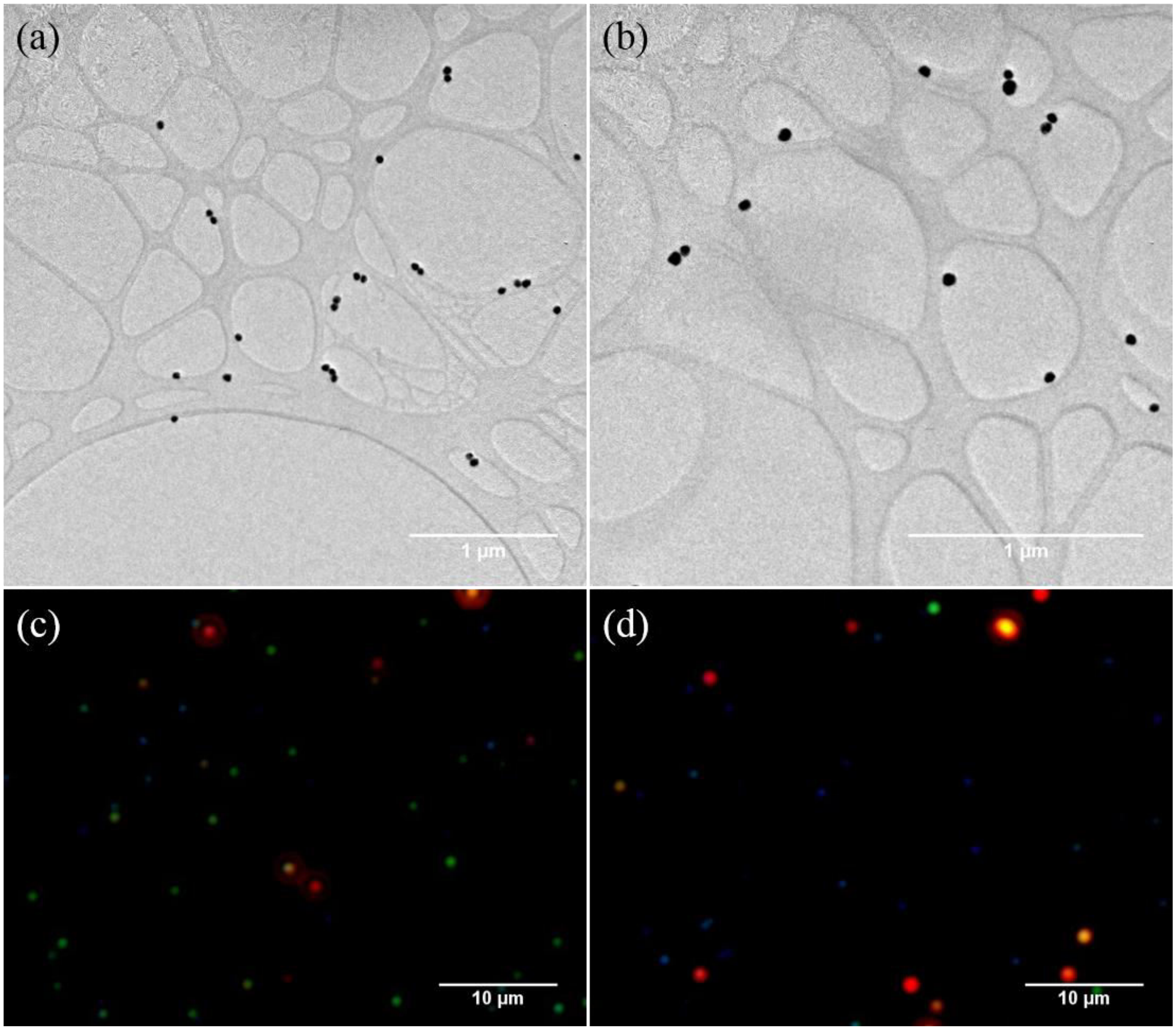

Supplementary Figure 3. Representative (a and b) low-magnification transmission electron micrographs (TEM) and (c and d) color-camera dark-field scattering images showing the low-area-density dispersion of the Ag NP sample accomplished by our drop-casting method. Each discrete scattering object resolved in the dark-field image is qualified as a single emitter in our studies, although the emitter may consist of a single NP or an aggregate of a handful of NPs. For (a) and (b), as-received Ag NP colloid was sonicated and drop-cast on a TEM grid (ultrathin carbon film on a lacey carbon film supported by a 400 mesh Cu grid, Ted Pella) and allowed to dry in a vacuum desiccator under heating at 80–90 °C for 5 h. For (c) and (d), the as-received Ag NP colloid (diluted x5) was dropcast on a glass coverslip and allowed to dry in a vacuum desiccator under heating at 80–90 °C for 5h. The sample-bearing coverslip was integrated into a flow cell for optical microscopy.

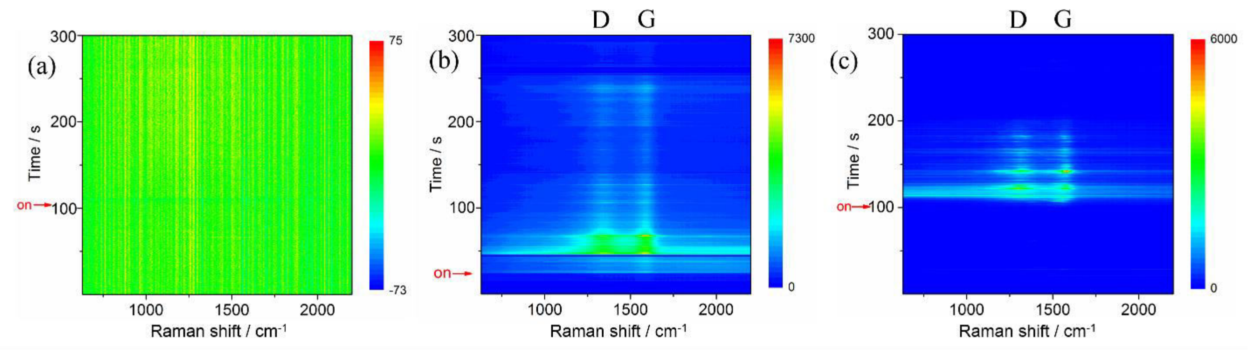

Supplementary Figure 4. Time series of unnormalized surface-enhanced Raman scattering (SERS) spectra, shown as waterfall plots for representative cases of (a) a non-responsive Ag NP emitter; (b) a Ag NP emitter that shows the formation of graphenic carbon, indicated by D and G Raman bands, and (c) a Ag NP emitter showing formation of both graphenic carbon and reactive species related to C_2H_4 oxidation species. Panels (a–c) correspond to the normalized plots shown in Figure 1 c–e, respectively. The instant when C_2H_4 flow was introduced is labeled as on.

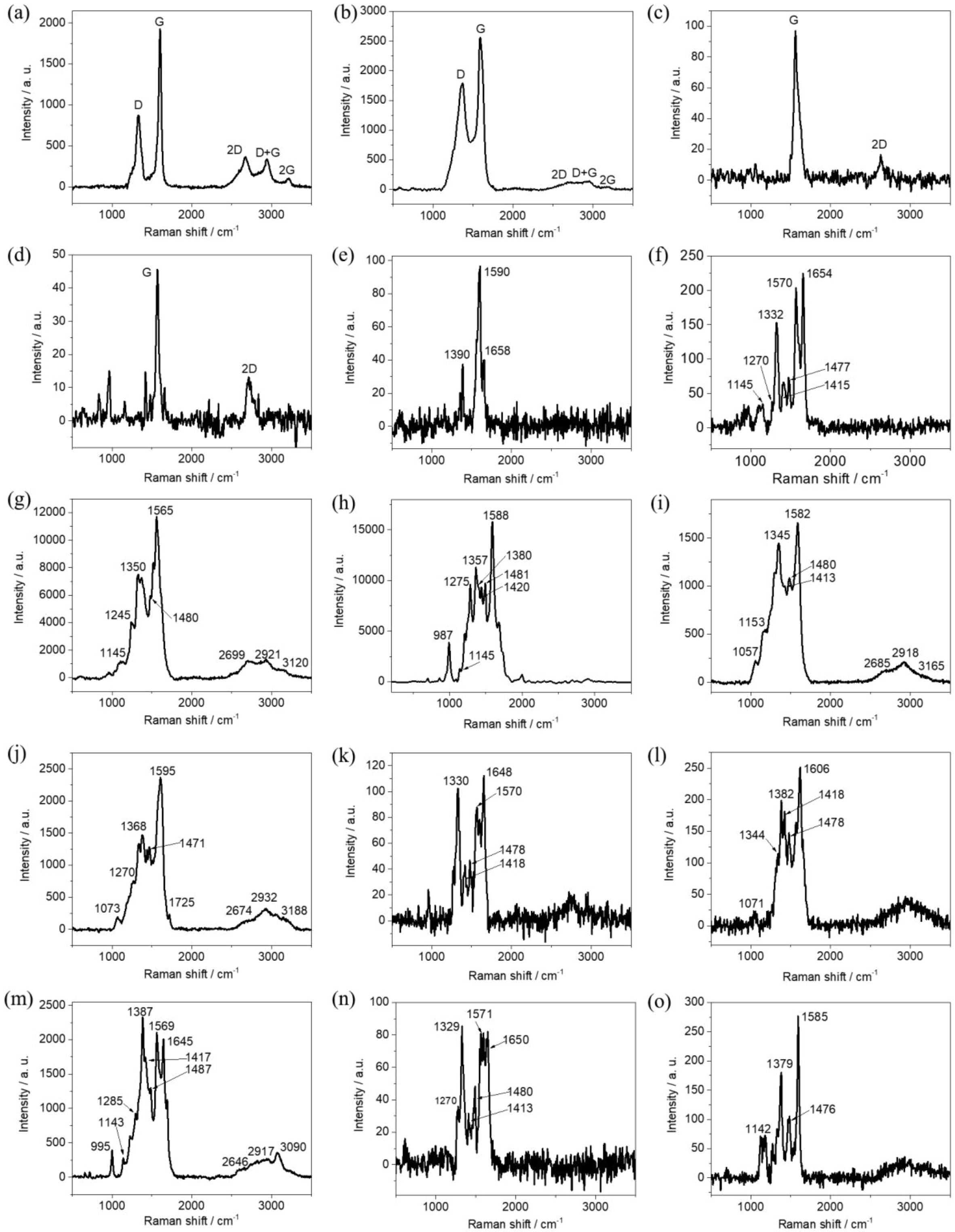

Supplementary Figure 5. More examples of single-frame SERS spectra that show modes corresponding to (a–e) presence of graphenic carbon and (f–o) presence of graphenic carbon and

C_2H_4 oxidation species. Modes are labeled by the peak wavenumber in cm^{-1}. The reader is referred to Supplementary Table 1 and Table 1 for mode assignments.

Most of these SERS spectra are from single-emitter studies of samples prepared using ca. 60 nm Ag NP colloid from Ted Pella. However, to ensure generality of our findings, we also examined SERS spectra from single-emitters prepared using another method.[15] Briefly, a mixture of 100 mL aqueous solution of 10 mM silver nitrate and 5 mL of aqueous 2 % (w/w) of 25 kDa PEI was heated to boiling for 15 min. The color of the solution gradually changed from dark yellow-brown to yellow. The reaction mixture was naturally allowed to cool down and a UV–Vis absorbance spectrum was collected, which showed an LSPR maximum at ~406 nm (Supplementary Figure 2). TEM shows Ag NPs of average size 28.3 nm ± 11.6 nm (Supplementary Figure 1 d–f) with considerable dispersion in size and shape. The as-synthesized Ag NP colloid was centrifuged at 6300X g, followed by washing with Nanopure water three times, re-dispersed in ~30 mL of Nanopure water, and stored in a refrigerator. Sample preparation, cleaning, and method for in situ SERS studies was identical to studies with Ted Pella colloids.

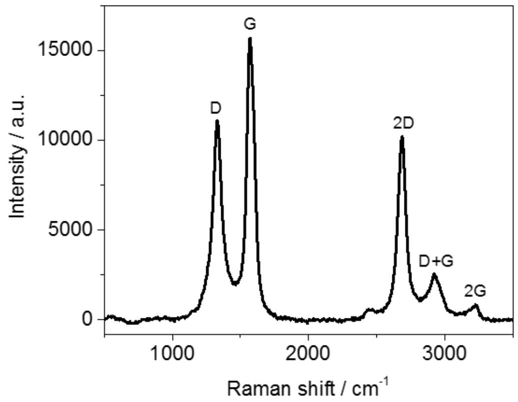

Supplementary Figure 6. A representative Raman spectrum of a graphene flake. A 10 μL solution of graphene in ethanol was drop-cast onto a glass coverslip and dried in air. The sample was excited by a focused 514.5 nm laser (5 mW, x60) and continuous Raman spectra were collected with an acquisition time of 30 s.

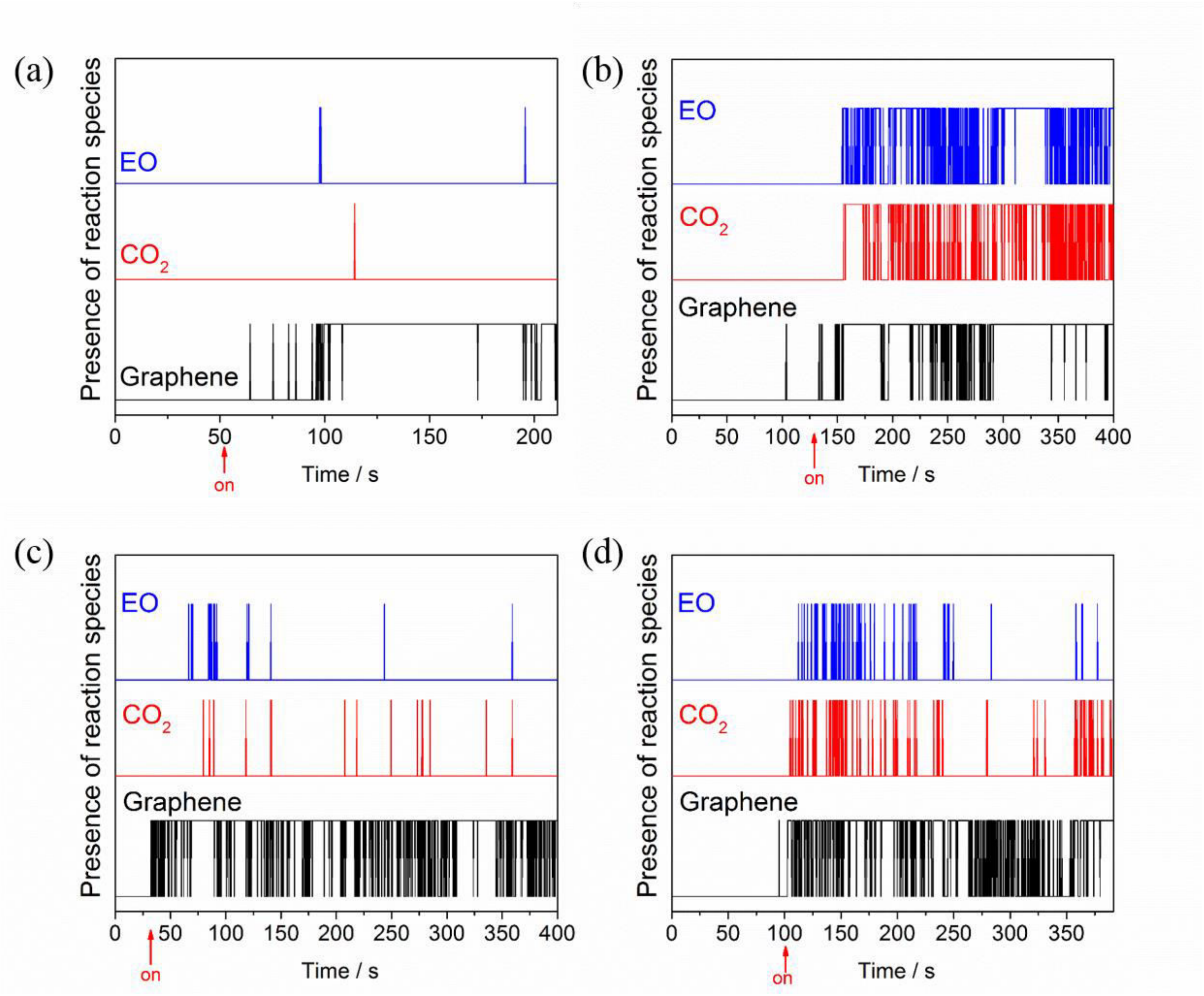

Supplementary Figure 7. To supplement the results shown in Figure 2a, four more examples of (a–d) digital reaction trajectories from four single Ag NP emitters are shown to demonstrate the correlated occurrence of graphenic carbon and C_2H_4 oxidation reaction species. Trajectories are shown vertically stacked. The instant when C_2H_4 flow was introduced is labeled as on.

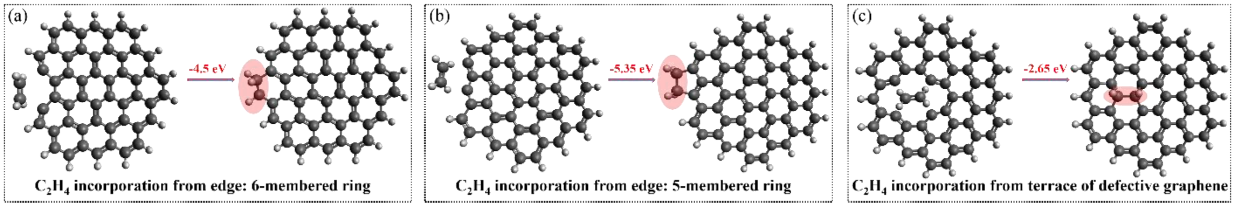

Supplementary Figure 8. DFT calculations of the energetics of C_2H_4 chemisorption onto a graphene nanofragment with (a and b) an edge defect and (c) terrace defect. The arrow signifies that a geometry optimization of the chemical species on the left leads to the species on the right. The free energy for the elementary step is indicated in eV atop each arrow. The highlighted regions indicate the chemisorption sites. Detailed free energy calculations are shown in Supplementary Note 3.

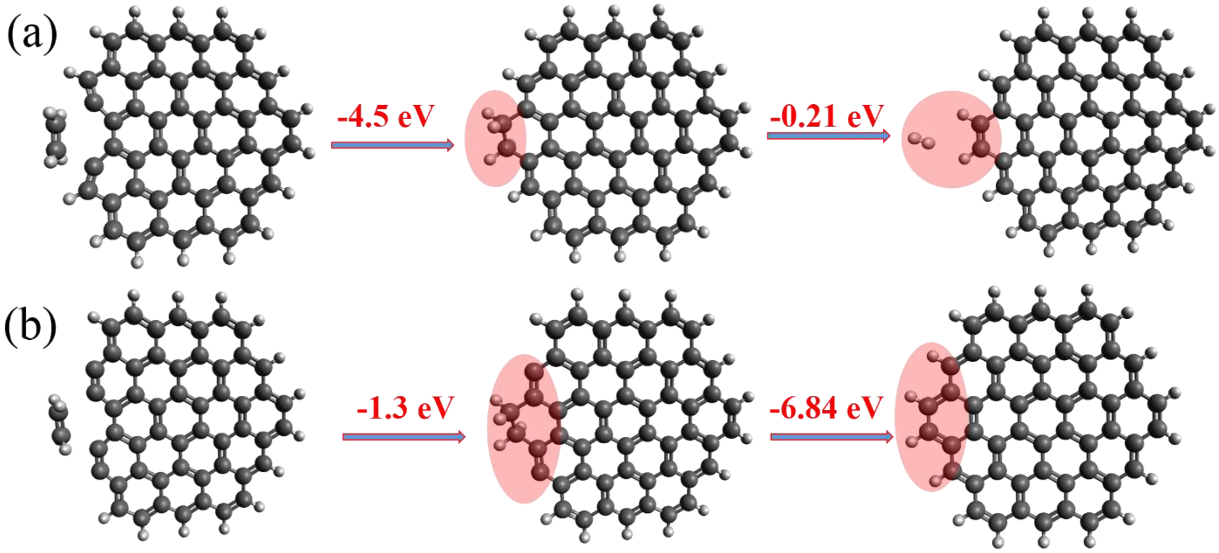

Supplementary Figure 9. For two types of graphene defects (a and b) DFT simulations demonstrate that following the incorporation of C_2H_4 into the defect (first step), the resulting structure releases strain (second step) by the breakage of two C-H bonds (as rendered in the simulation) and the release of H_2. The arrow signifies that a geometry optimization of the chemical species on the left leads to the species on the right. The free energy for the elementary step is indicated in eV atop each arrow. The highlighted regions indicate the chemisorption sites. Note the first step in (a) is the same data as that in Supplementary Figure 8a. Detailed free energy calculations are shown in Supplementary Note 3.

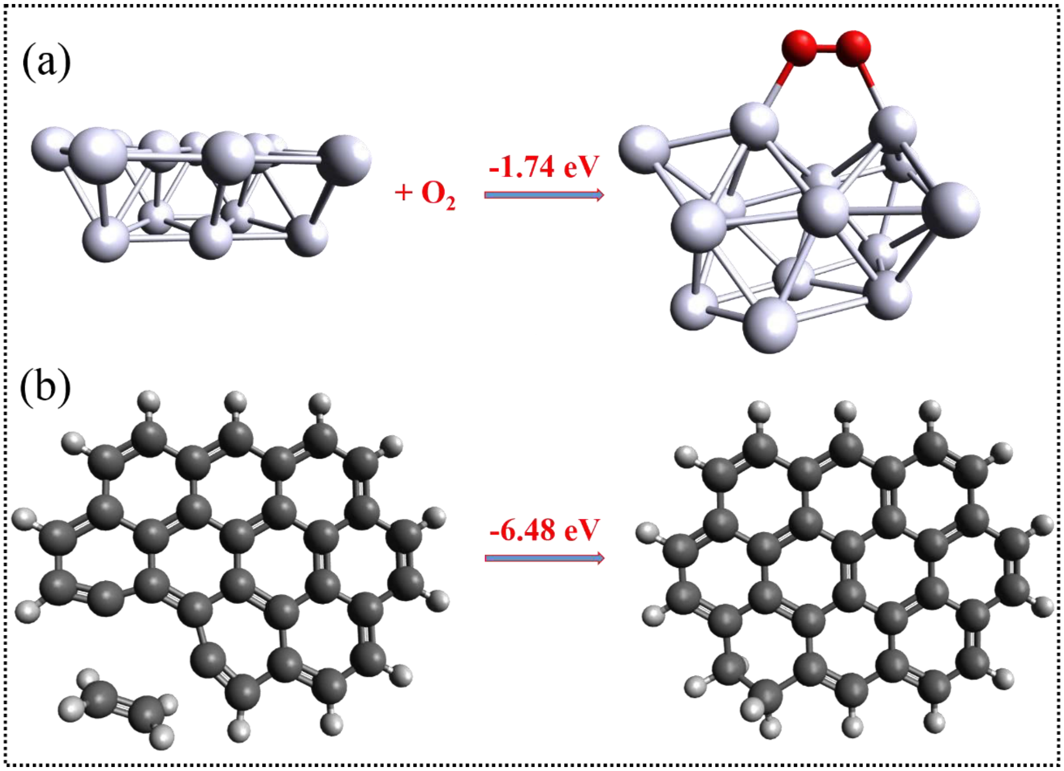

Supplementary Figure 10. DFT-computed energetics of (a) O_2 adsorption on Ag (111) surface of the model Ag cluster; (b) C_2H_4 chemisorption onto an edge defect of graphene. The arrow signifies that a geometry optimization of the chemical species on the left leads to the species on the right. O atoms are shown in red, C atoms in dark gray, and H atoms in light gray, similar to Figure 4. Ag atoms are shown in white. The free energy for the elementary step is indicated in eV atop each arrow. Detailed free energy calculations are shown in Supplementary Note 3.

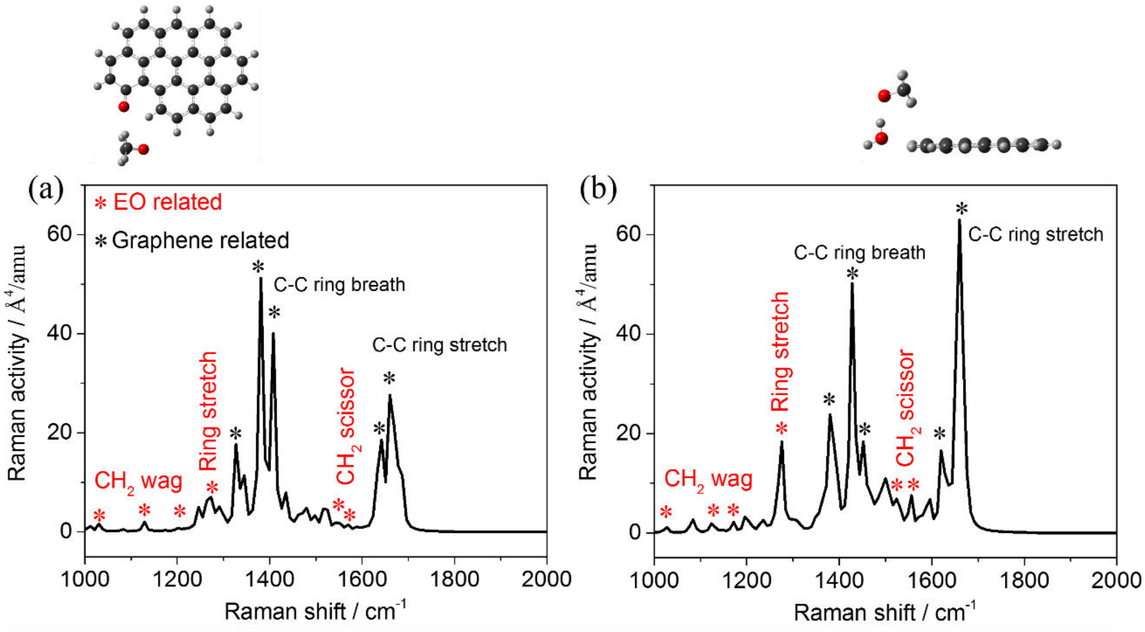

Supplementary Figure 11. DFT-computed Raman activities of the products of the two reaction pathways in Figure 4: (a) VI and (b) IX, optimized geometries of which are shown above the spectra. Key vibrational modes of EO (red) and graphenic carbon (black) are highlighted.

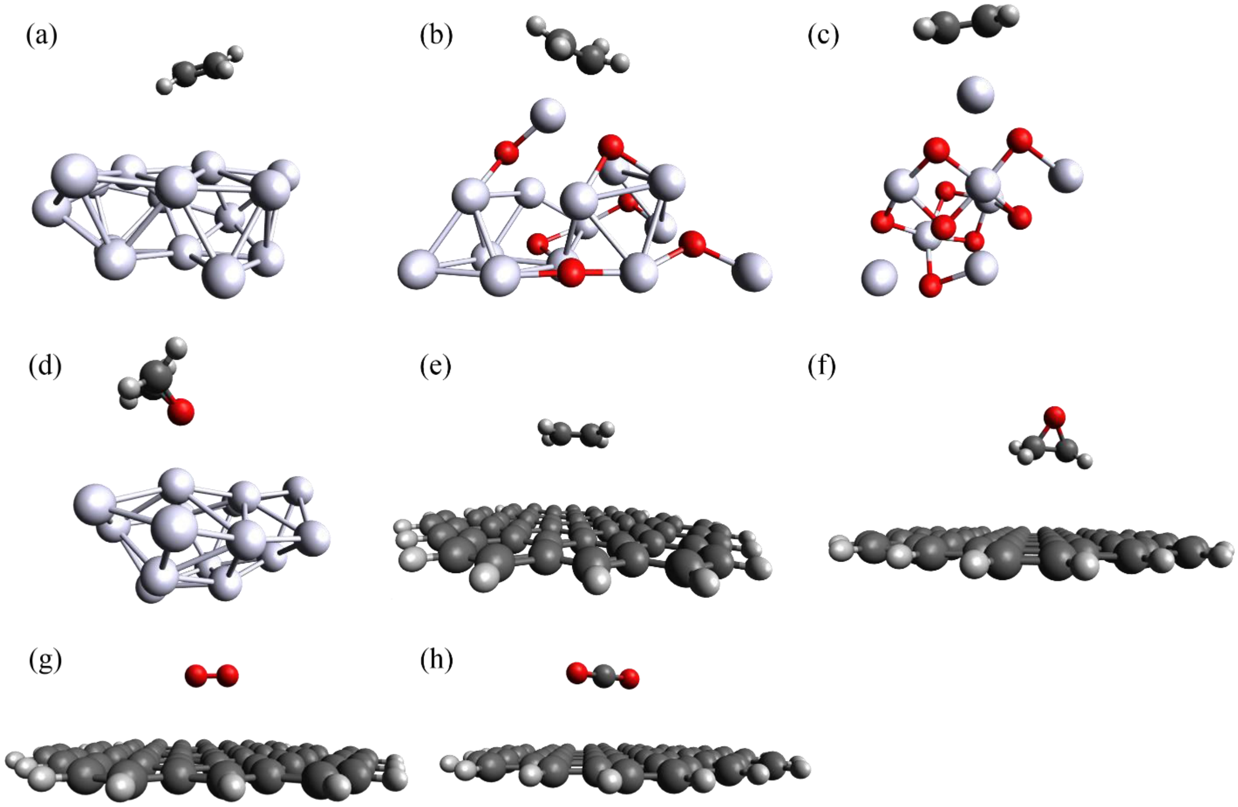

Supplementary Figure 12. Adsorption of reaction species on different surfaces. DFT-optimized geometries for C_2H_4 adsorption on (a) Ag(111) surface, (b) Ag_2O and (c) AgO model clusters; (d) EO adsorption on Ag (111) surface; (e) C_2H_4 adsorption on defect-free graphene; (f) EO adsorption on defect-free graphene; (g) O_2 adsorption on defect-free graphene; (h) CO_2 adsorption on defect-free graphene. Supplementary Table 2 summarizes the free energy of adsorption for each case. Only cases (c) and (d) are found to be exergonic. Detailed free energy calculations are shown in Supplementary Note 3.

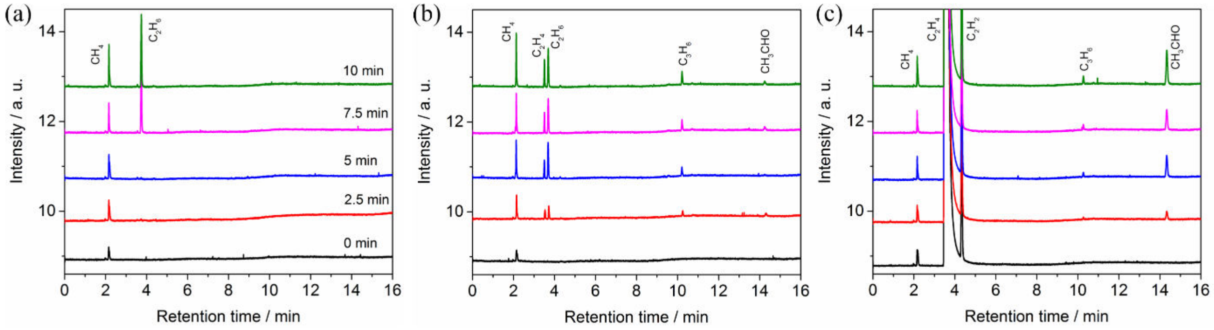

Supplementary Figure 13. Gas chromatograms (GC) as a function of reaction time (stacked vertically) for three control photoreactions involving: (a) Ag/Al_2O_3 in air, (b) G-Ag/Al_2O_3 in air, and (c) G/Al_2O_3 in C_2H_4 and air. No production of ethylene oxide (EO) was observed in any of these cases. We did observe hydrocarbon byproducts, potentially generated by photodegradation of carbon contaminants in the sample and/or graphene. Acetaldehyde (CH_3CHO) was also observed in the control experiments with graphene in (b) and (c), possibly from photo-oxidation of graphene. All tests were carried out in batch mode under 514.5 nm excitation (0.5 W, 2 mm beam diameter). For each type of control, two independent trials were run, results from one of which are shown here. GC peaks were labeled on the basis of retention times determined from standard runs of EO, acetaldehyde, and hydrocarbon mixture.

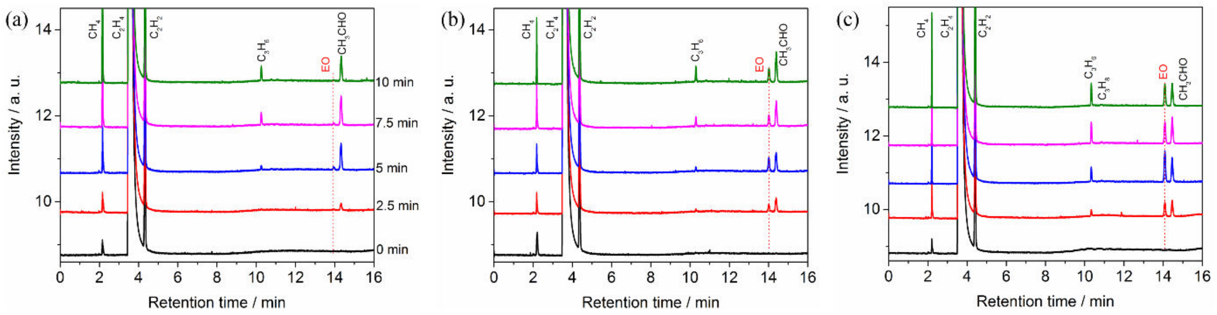

Supplementary Figure 14. Gas chromatograms as a function of reaction time (stacked vertically) for three ethylene photo-epoxidation reactions conducted in C_2H_4 and air for: (a) Ag/Al_2O_3, (b) A-Ag/Al_2O_3, and (c) G-Ag/Al_2O_3 catalysts. EO generation was seen as a function of reaction time for A-Ag/Al_2O_3 and G-Ag/Al_2O_3 catalysts, but negligible to no EO production is seen for Ag/Al_2O_3. Note, we also observe hydrocarbon byproducts, deemed from the control experiments shown in Supplementary Figure 13, to be from photo-degradation of carbon contaminants in the sample and/or graphene. CH_3CHO was also observed, which may be from photo-degradation or from isomerization of EO.[16] Under the ambient temperature and atmospheric conditions of our photocatalytic reaction, full oxidation of CH_3CHO to CO_2 can be expected to be slower than in the case of conventional thermal C_2H_4 epoxidation, wherein any CH_3CHO formed is rapidly oxidized to CO_2. All tests were carried out in batch mode under 514.5 nm excitation (0.5 W, 2 mm beam diameter). For each type of control, 3–4 independent trials were run, results from one of which are shown here. GC peaks were labeled on the basis of retention times determined from standard runs of EO, acetaldehyde, and hydrocarbon mixture.

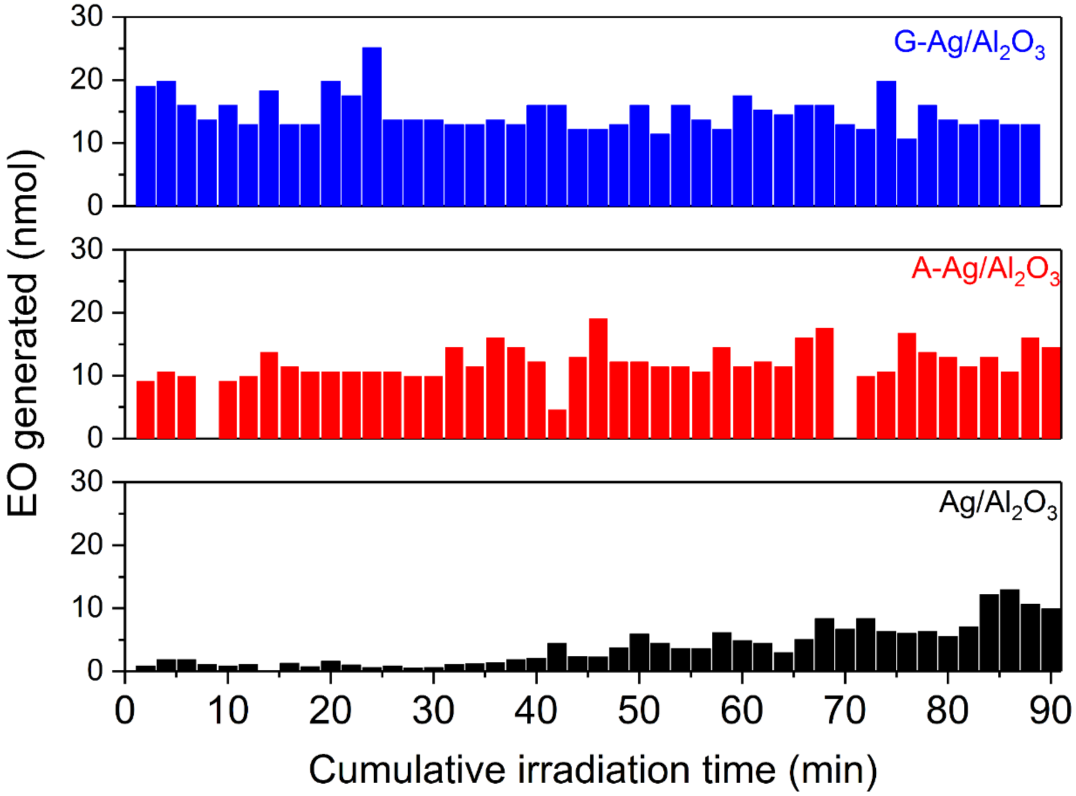

Supplementary Figure 15. Amount of EO generated in photocatalytic ethylene oxidation, under quasi-steady-state reaction conditions, using three photocatalysts: (top row) G-Ag/Al_2O_3, (middle row) A-Ag/Al_2O_3, and (bottom row) Ag/Al_2O_3. The photocatalytic reaction was carried out over a cumulative 90 min period, consisting of multiple 2 min cycles. In each cycle, the catalyst powder (15 mg) was irradiated by a 0.5 W, 514.5 nm laser in the presence of a set concentration of ethylene and air. The EO generated in the head-space after each 2 min cycle was measured by GC-FID, which is plotted here in nmol units. The G-Ag/Al_2O_3 and A-Ag/Al_2O_3 photocatalysts appear to sustain their photo-epoxidation activity over multiple cycles of operation spanning the 90 min photocatalytic reaction. The epoxidation activity averaged across the entire time-span is plotted in Figure 3a for G-Ag/Al_2O_3 and A-Ag/Al_2O_3 photocatalysts. The Ag/Al_2O_3 photocatalyst, which exhibits little to no low activity over the first 30 min, appears to approach an activated state, as deemed from its progressively increasing photo-epoxidation activity beyond 30 min. The activity of the Ag/Al_2O_3 photocatalyst averaged over the first 30 min, prior to activation, is plotted in Figure 3a.

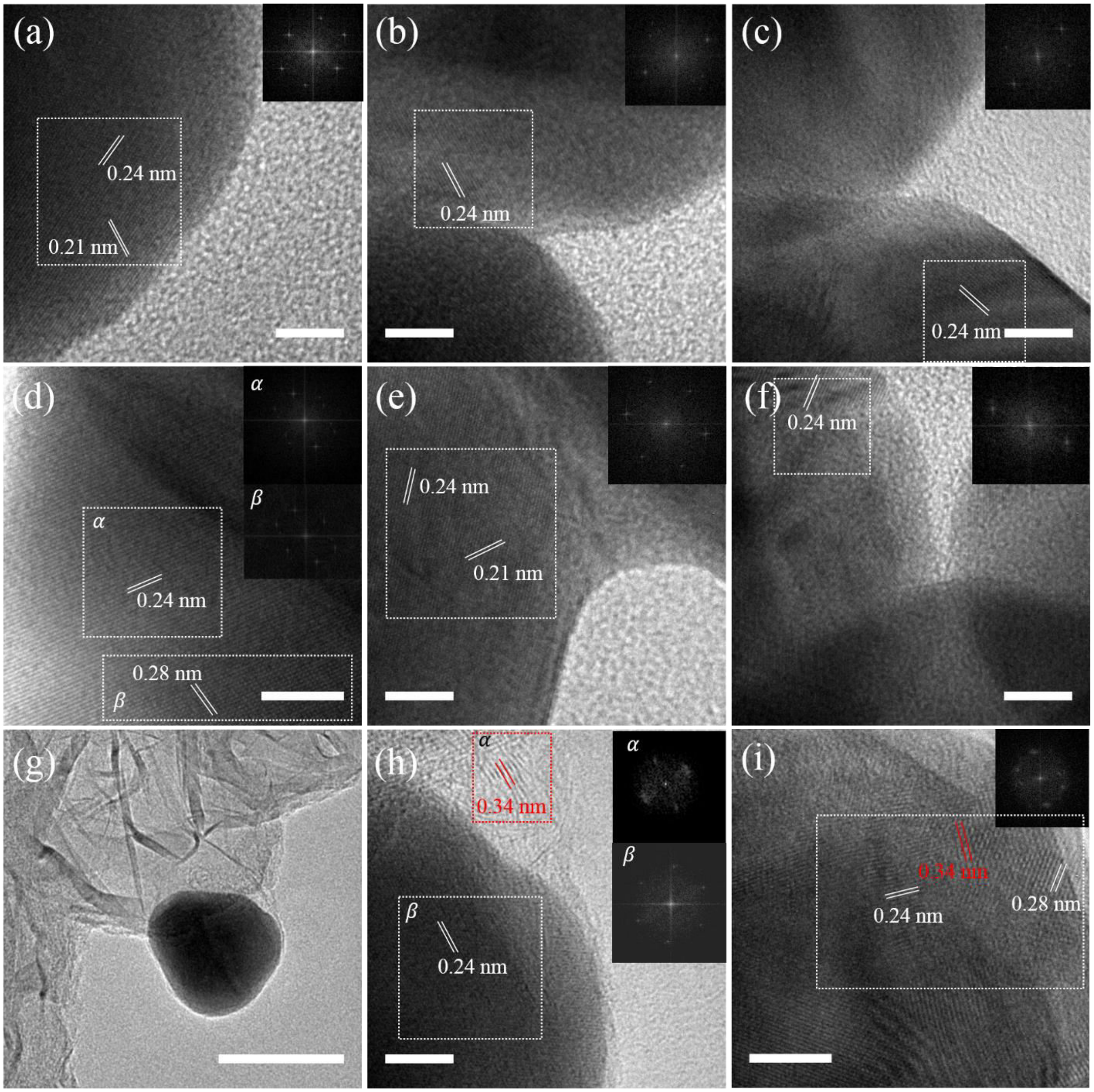

Supplementary Figure 16. Representative high-resolution transmission electron microscopy (HRTEM) images of (a–c) Ag NPs from Ted Pella, (d–f) Ag NPs irradiated by a 514.5 nm laser (5 mW, x60) in ambient air and (g–i) Ag NPs covered by commercial graphene. Lattice spacings indicated were obtained by a fast-Fourier transform (FFT) of a selected region (coarsely marked by a square and labeled as α or β) of the image. These FFTs, following background subtraction, are shown in the insets. Lattice spacings indicated in white font correspond to Ag (0.24 nm and 0.21 nm) and those indicated in red font correspond to graphene (0.34 nm). Except for g, where the scale bar represents 50 nm, all panels have a scale bar representing 5 nm. The HRTEM sample preparation is described in the Methods section.

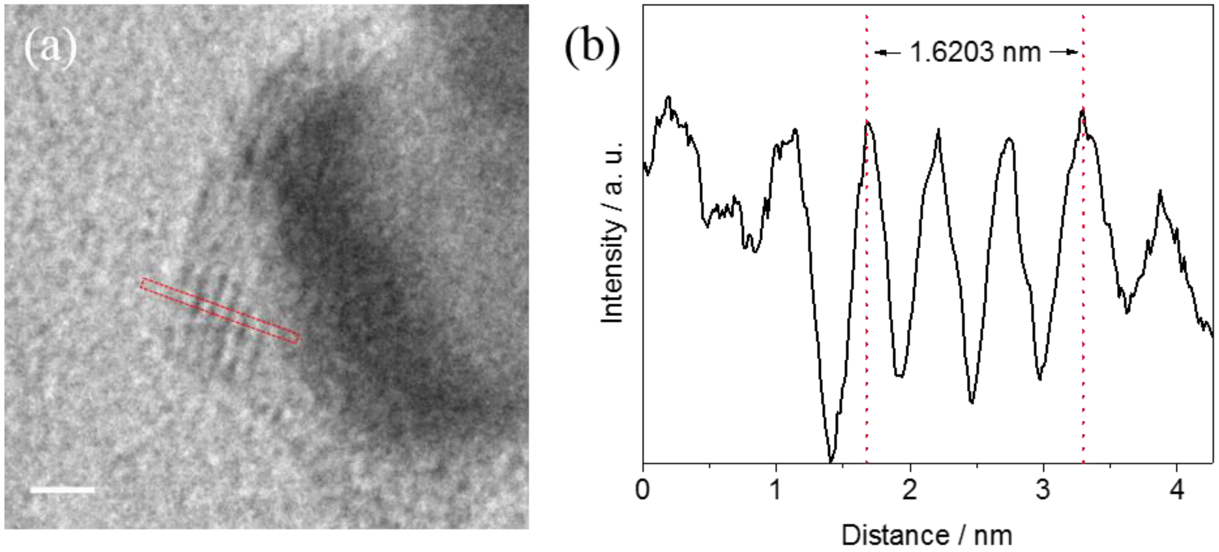

Supplementary Figure 17. (a) Representative HRTEM image of Ag NPs irradiated with 514.5 nm laser (5 mW, x60) in C_2H_4 showing the presence of defective or partially oxidized graphene with an inter-layer spacing of ~0.54 nm as determined by the (b) profile taken along a line, drawn perpendicular to the lattice fringes. The line profile was generated in Gatan Digital Micrograph software. The profile shown in (b) is an averaged result across the selected region coarsely marked in (a). The scale bar in (a) represents 2 nm.

Supplementary Notes

1. Estimation of turnover frequency (TOF) for EO generation:

Calibration of GC peak area for EO: The volume of 1 mol of ideal gas at room temperature is ~24.5 L ($V = n\mathrm{R}T/P$). The concentration of EO calibration standard gas is 100 ppm, which amounts to 10^{-4} mol per 24.5 L = $4.1*10^{-6}$ mol L^{-1}. Based on a linear fit to the calibration curve (EO GC peak area vs. injection volume):

$$y = 0.0485 * x$$

where x is the injection volume in μL and y is integrated peak area of EO.

Using this calibration information, the amount of product EO in the reactor headspace can be quantified from the measured GC peak area as:

$$\text{Amount of EO produced} = 4.1*10^{-6}\ \text{mol L}^{-1} * 10^{-6}\ \text{L}\ \mu\text{L}^{-1} * y/0.0485\ \mu\text{L} * 90 = 7.61 * y\ \text{nmol}$$

The factor 90 accounts for the fact that the total reactor headspace volume is 9 mL, which is 90-fold the gas volume sampled for GC-FID analysis.

Estimation of number of Ag NPs: The catalyst powder was estimated to be a pellet of diameter of 6 mm. Therefore, the volume of 15 mg catalyst powder was estimated to be ~$4/3 * \pi * 0.003^3$ m^3. Not all the Ag NPs within the catalyst powder fall within the irradiation zone of the laser. The laser beam diameter is ~2 mm, the penetration depth of the laser is ~0.5 mm. From these estimates, the volume of working catalyst is estimated to be $\pi * 0.001^2 * 0.0005$ m^3. Therefore, ~0.014 * 15 mg = 0.21 mg of the overall (15 mg) catalyst is actually within the irradiation zone and therefore constitutes the working fraction of the catalyst. The loading of Ag is 1:4 by weight of Al_2O_3. Therefore, the working mass of Ag is ~0.042 mg.

Assuming a NP diameter of 30 nm (as estimated from the LSPR maximum in UV–Vis absorbance spectrum), the volume of one Ag NP is $4/3 * \pi * 15^3 = 14100$ nm^3. Based on the density of Ag of 10.5 g cm^{-3}, the mass of a Ag NP is $14100 * (10^{-7})^3 * 10.5$ g = 1.48×10^{-21} g. The number of working Ag NPs = mass of working Ag/mass of a Ag NP = $0.042 * 10^{-3}$ g/$1.48 * 10^{-16}$ g = $2.84 * 10^{11}$

TOF for EO generation is estimated as:

$$\text{TOF} = \text{moles of EO generated per s} * N_A / \text{number of working Ag NPs}$$

where N_A is Avogadro's number. Therefore,

$$\text{TOF} = \text{nmoles of EO generated per s} * 2120$$

2. Estimation of quantum yield

In the quasi-steady state bulk photocatalytic reactions, we employ a laser power of 0.5 W and a wavelength of 514.5 nm. Since the laser beam is fully incident on the catalysts powder and also fully absorbed by the catalyst sample, absorbed laser power = 0.5 W or 0.5 J s^{-1}.

Energy of each photon at 514.5 nm = $3.86 * 10^{-19}$ J

Number of photons absorbed per s, N_{ph} = $(0.5 * 10^{19}/3.86)$ s^{-1} = $1.29 * 10^{18}$ s^{-1}

Quantum yield = TOF (NP^{-1} s^{-1}) * Estimated number of working NPs / ($1.29 * 10^{18}$)

For the G-Ag/Al_2O_3 catalyst, TOF = 262 NP^{-1} s^{-1}

Hence, its quantum yield = $262 * 2.84 * 10^{11}$ / ($1.29 * 10^{18}$) = $5.75 * 10^{-5}$ EO molecules generated per photon.

3. Records of all free energy calculations

Note: G means the result of (ε_o + G_{corr}); 1 Hartree = 27.2116 eV

Supplementary Table 3. Free energy calculations for Figure 4

Name of species	(ε_o + G_{corr}), Hartree
I	-1149.689130
II	-1300.3140
III	-1300.4230
IV	-1378.931587
V	-1379.565209
VI	-1379.48698
VII	-1378.913389
VIII	-1380.1200
IX	-1381.285477
O_2	-150.274864
Ag_{15}	-2187.18
Ag_{15}^+	-2187.00
C_2H_4	-78.561123
H atom	-0.51

Free energies of elementary steps in Pathway A:

Free energy of reaction from I to II: G(II) - G(I) - G(O_2)

= -(1300.314 - 150.274864 - 1149.689130) Hartree = -0.350006 Hartree = -9.52 eV

Free energy of reaction from II to III: G(III) + G(Ag_{15}^+) - G(II) - G(Ag_{15})

= -(2187 **+** 1300.423 - 1300.314 - 2187.18) Hartree = 0.07055 Hartree = 1.92 eV

Free energy of reaction from III to IV: G(IV) - G(III) - G(C_2H_4)

= -(1378.931587 - 78.561123 - 1300.42) Hartree = 0.04985208 Hartree = 1.36 eV

Free energy of reaction from IV to V: G(V) - G(IV) - G(H)

= -(1379.565209 - 0.51 - 1378.931587) Hartree = -0.1232545067476164 Hartree = -3.35 eV

Free energy of reaction from V to VI: G(VI) + G(Ag_{15}) - G(V) - G(Ag_{15}^+)

= -(1379.486983 **+** 2187.18 - 2187 - 1379.565209) Hartree = -0.1 Hartree = -2.72 eV

Free energies of elementary steps in Pathway B:

Free energy of reaction from I to II: G(II) - G(I) - G(O_2)

= -(1300.314 - 150.274864 - 1149.689130) Hartree = -0.3519849 Hartree = -9.52 eV

Free energy of reaction from II to III: G(III) + G(Ag_{15}^+) - G(II) - G(Ag_{15})

= -(2187 + 1300.423 - 1300.314 - 2187.18) Hartree = 0.07055 Hartree = 1.92 eV

Free energy of reaction from III to IV: G(IV) - G(III) - G(C_2H_4)

= -(1378.931587 - 78.561123 - 1300.42) Hartree = 0.04985208 Hartree = 1.36 eV

Free energy of reaction from IV to VII: G(VII) + G(Ag_{15}) - G(Ag_{15}^+) + G(IV)

= -(2187.185 + 1378.913389 - 1378.931587 - 2187) Hartree = - 0.1660670134958 Hartree = -4.52 eV

Free energy of reaction from VII to VIII: G(VIII) + G(Ag_{15}^+) - G(VII) - 2*G(H) - G(Ag_{15})

= -(2187 + 1380.12 - 1378.913389 - 2187.185 - 1.02) Hartree = -0.005144906 Hartree = -0.14 eV

Free energy of reaction from VIII to IX: G(IX) + G(Ag_{15}) - G(Ag_{15}^+) - G(VIII) - 2*G(H)

= -(2187.185 + 1381.285477 - 1380.12 - 2187-1.02) = - 0.33010950674839 = -8.98 eV

Supplementary Table 4. Free energy calculations for Supplementary Figure 8

Name of species	(ε_o + G_{corr}), Hartree
Defective graphene (edge), Panel a left	-1990.433378
C_2H_4 incorporated in defective graphene, Panel a right	-2069.160837
Defective graphene (edge), Panel b left	-2066.592887
C_2H_4 incorporated defective graphene, Panel b right	-2145.351657
Defective graphene (terrace), Panel c left	-1991.554803
C_2H_4 incorporated in defective graphene, Panel c right	-2070.214289
C_2H_4	-78.561123

a. G(Panel a_R) - G(Panel a_L) - G(C_2H_4)

   = -(2069.160837 - 1990.433378 - 78.562123) = -0.165336 Hartree = -4.5 eV

b. G(Panel b_R) - G(Panel b_L) - G(C_2H_4)

   = -(2145.351657 - 2066.592887 - 78.562123) Hartree = -0.196647 Hartree = -5.35 eV

c. G(Panel c_R) - G(Panel c_L) - G(C_2H_4)
   = -(2070.214289 - 1991.554803 - 78.562123) Hartree = -0.097363 Hartree = -2.65 eV

Note: R, M, and L represent reaction species on right, middle or left column of the figure.

Supplementary Table 5. Free energy calculations for Supplementary Figure 9

Name of species	(ε_o + G_{corr}), Hartree
Defective graphene, Panel a left	-1990.433378
C_2H_4 incorporated in defective graphene, Panel a middle	-2069.160837
Strain-relieved graphene, Panel a right	-2067.991872
Defective graphene, Panel b left	-1989.09517
C_2H_4 incorporated in defective graphene, Panel b middle	-2067.7405066
Strain-relieved graphene, Panel b right	-2067.991872
C_2H_4	-78.561123
H_2	-1.176824

a. G(Panel a_M) - G(Panel a_L) - G(C_2H_4)

= -(2069.160837 - 1990.433378 - 78.562123) Hartree = -0.165336 Hartree = -4.5 eV

G(Panel a_R)+G(H_2)-G(Panel a_M)

= -(2067.991872 + 1.176824 - 2069.16083) Hartree = -0.007866 Hartree = -0.21 eV

b. G(Panel a_M) - G(Panel a_L) - G(C_2H_4)

= -(2067.7405066 - 1989.09517 - 78.562123) Hartree = -0.0832136Hartree = -1.3 eV

G(Panel a_M) - G(Panel a_L)

= -(2067.991872 - 2067.7405066) Hartree = -0.2513654 Hartree = -6.84 eV

Supplementary Table 6. Free energy calculations for Supplementary Figure 10

Name of species	(ε_o + G_{corr}), Hartree
Ag_{15}	-2187.184866
Ag_{15} with O_2 incorporated	-2337.523554
Defective graphene, Panel b left	-1149.689130
C_2H_4 incorporated in defective graphene, Panel b right	-1228.489424
C_2H_4	-78.561123
O_2	-150.274864

a. G(Panel a_R) - G(Panel a_L) - G(O_2)

= -(2337.523554 - 150.274864 - 2187.184866) Hartree = -0.063824 Hartree = -1.74 eV

b. G(Panel b_R) - G(Panel b_L) - G(C_2H_4)

= -(1228.489424 - 1149.689130 - 78.562123) Hartree = -0.2381356 Hartree = -6.48 eV

Supplementary Table 7. Free energy calculations for Supplementary Figure 12

Name of species	($\varepsilon_o + G_{corr}$), Hartree
Ag_{15}	-2187.184866
Ag_{15} with C_2H_4 adsorbed	-2265.721430
Ag_2O cluster	-2492.551837
Ag_2O cluster with C_2H_4 adsorbed	-2571.088932
AgO cluster	-1767.644072
AgO cluster with C_2H_4 adsorbed	-1846.244266
EO	-153.696659
Ag_{15} with EO adsorbed	-2340.901040
Non-defective graphene	-2067.986236
C_2H_4 on non-defective graphene	-2146.515593
EO on non-defective graphene	-2221.670255
O_2 on non-defective graphene	-2218.198597
CO_2 on non-defective graphene	-2256.488925
C_2H_4	-78.561123
O_2	-150.274864

a. $G(Ag_{15} + C_2H_4) - G(Ag_{15}) - G(C_2H_4)$
   = -(2265.721430 - 78.562123 - 2187.184866) Hartree = 0.0255223 Hartree = 0.69 eV

b. $G(Ag_2O + C_2H_4) - G(Ag_2O) - G(C_2H_4)$
   = -(2571.088932 - 78.562123 - 2492.551837) Hartree = 0.025 Hartree = 0.68 eV

c. $G(AgO + C_2H_4)\text{-}G(AgO)\text{-}G(C_2H_4)$
   = -(1846.244266 - 78.562123 - 1767.644072) Hartree = -0.038071 Hartree = -1.04 eV

d. $G(Ag_{15} + EO) - G(Ag_{15}) - G(EO)$
   = -(2340.901040 - 153.696659 - 2187.184866) Hartree = -0.019515 Hartree = -0.53 eV

e. $G(Graphene + C_2H_4) - G(Graphene) - G(C_2H_4)$
   = -(2146.515593 - 78.562123 - 2067.986236) Hartree = 0.032766 Hartree = 0.89 eV

f. $G(Graphene + EO) - G(Graphene) - G(EO)$
   = -(2221.670255 - 153.696659 - 2067.986236) Hartree= 0.01264 Hartree = 0.34 eV

g. $G(Graphene + O_2) - G(Graphene) - G(O_2)$
   = -(2218.199260 - 150.274864 - 2067.986236) Hartree = 0.062503 Hartree = 1.7 eV

h. $G(Graphene + CO_2) - G(Graphene) - G(CO_2)$
   = -(2256.488925 - 2067.986236 - 188.510369) Hartree = 0.00768 Hatree = 0.21 eV

Supplementary References

1. Roeges, N. P. G. A guide to the complete interpretation of infrared spectra of organic structures (John Wiley, New York, 1994).
2. Castro, J. L., Otero, J. C. & Marcos, J. I. Anomalous SERS of monocarboxylic acids on silver sols. *J. Raman Spec.* **28**, 765–769 (1997).
3. Liu, H. T., Ryu, S. M., Chen, Z. Y., Steigerwald, M. L., Nuckolls, C. & Brus, L. E. Photochemical reactivity of graphene. *J. Am. Chem. Soc.* **131**, 17099–17101 (2009).
4. Ferrari, A. C. Raman spectroscopy of graphene and graphite: Disorder, electron-phonon coupling, doping and nonadiabatic effects. *Solid State Comm.* **143**, 47–57 (2007).
5. Kang, L. L., Chu, J. Y., Zhao, H. T., Xu, P. & Sun, M. T. Recent progress in the applications of graphene in surface-enhanced Raman scattering and plasmon-induced catalytic reactions. *J. Mater. Chem. C* **3**, 9024–9037 (2015).
6. Moskovits, M. & Dilella, D. P. Enhanced Raman-spectra of ethylene and propylene adsorbed on silver. *Chem. Phys. Lett.* **73**, 500-505 (1980).
7. Dilella, D. P. & Moskovits, M. Surface-enhanced Raman-spectra of some butenes adsorbed on silver. *J. Phys. Chem.* **85**, 2042–2046 (1981).
8. Patterson, M. L. & Weaver, M. J. Adsorption and oxidation of ethylene at gold electrodes as examined by surface-enhanced Raman pectroscopy. *J. Phys. Chem.* **89**, 1331–1334 (1985).
9. Linic, S. & Barteau, M. A. Formation of a stable surface oxametallacycle that produces ethylene oxide. *J. Am. Chem. Soc.* **124**, 310–317 (2002).
10. Maynard, K. J. & Moskovits, M. A surface enhanced Raman study of carbon dioxide coadsorption with oxygen and alkali metals on silver surfaces. *J. Chem. Phys.* **90**, 6668–6679 (1989).
11. Wang, C. B., Deo, G. & Wachs, I. E. Interaction of polycrystalline silver with oxygen, water, carbon dioxide, ethylene, and methanol: In situ Raman and catalytic studies. *J. Phys. Chem. B* **103**, 5645–5656 (1999).
12. Shiraishi, Y. *et al.* Highly selective production of hydrogen peroxide on graphitic carbon nitride (g-C_3N_4) photocatalyst activated by visible light. *ACS Catal.* **4**, 774–780 (2014).
13. Kim, E. *et al.* Heme/Cu/O_2 reactivity: Change in Fe^{III}-(O_2^{2-})Cu^{II} unit peroxo binding geometry effected by tridentate copper chelation. *J. Am. Chem. Soc.* **126**, 12716–12717 (2004).
14. Kotz, R. & Yeager, E. Raman studies of the silver-silver oxide electrode. *J. Electroanal. Chem.* **111**, 105–110 (1980).
15. Kim, K., Lee, H. B., Lee, J. W. & Shin, K. S. Poly(ethylenimine)-stabilized silver nanoparticles assembled into 2-dimensional arrays at water–toluene interface. *J. Colloid Interface Sci.* **345**, 103–108 (2010).
16. Kenson, R. E. & Lapkin, M. Kinetics and mechanism of ethylene oxidation. Reactions of ethylene and ethylene oxide on a silver catalyst. *J. Phys. Chem.* **74**, 1493–1502 (1970).
